# Phosphorylation of the androgen receptor at Ser81 is co‐sustained by CDK1 and CDK9 and leads to AR‐mediated transactivation in prostate cancer

**DOI:** 10.1002/1878-0261.12968

**Published:** 2021-05-03

**Authors:** XinTao Gao, Jiaqian Liang, LiYang Wang, Zhaoyang Zhang, Penghui Yuan, Jiaxin Wang, Yanfei Gao, Fen Ma, Carla Calagua, Huihui Ye, Olga Voznesensky, Shaogang Wang, Tao Wang, Jihong Liu, Shaoyong Chen, Xiaming Liu

**Affiliations:** ^1^ Department of Urology Tongji Hospital Tongji Medical College Huazhong University of Science and Technology Wuhan China; ^2^ Hematology‐Oncology Division Department of Medicine Beth Israel Deaconess Medical Center Harvard Medical School Boston MA USA; ^3^ Wuhan No. 1 Hospital Tongji Medical College Huazhong University of Science and Technology Wuhan China; ^4^ Department of Biochemistry and Molecular Biology Institute of Basic Medical Sciences College of Basic Medicine Hubei University of Medicine Shiyan China; ^5^ Department of Orthopaedic Surgery The Second Affiliated Hospital Chongqing Medical University China; ^6^ Department of Pathology Beth Israel Deaconess Medical Center Harvard Medical School Boston MA USA; ^7^ Department of Pathology University of California Los Angeles CA USA

**Keywords:** androgen receptor, CDK1, CDK9, ChIP‐Seq, enhancer‐promoter loop, serine 81 phosphorylation

## Abstract

Androgen receptor (AR) is the principal molecule in prostate cancer (PCa) etiology and therapy. AR re‐activation still remains a major challenge during treatment of castration‐resistant prostate cancer (CRPC) tumors that relapse after castration therapies. Recent reports have indicated the enrichment of Ser81‐phosphorylated AR (pS81) in the nucleus of CRPC cells, and CDK1 and CDK9 as the kinases phosphorylating AR at S81. In the current study we showed that pS81 is preferentially localized in the nucleus in both rapid biopsy metastatic CRPC samples and PCa xenografts, and nuclear pS81 localization is correlated with AR transactivation in tumor xenografts. Chromatin immunoprecipitation (ChIP) analysis demonstrated an alignment of S81 phosphorylation and AR‐mediated transactivation with the chromatin locus openness. Moreover, pS81‐specific ChIP‐Seq showed a disproportional occupancy of pS81 on AR‐activated promoters, while 3C‐ChIP assays further indicated an enrichment of pS81 at the *PSA* enhancer‐promoter loop, a known AR activating hub. In the latter, CDK9 was shown to modulate the transactivation of the AR and RNA Pol II. Indeed, ChIP and re‐ChIP assays also confirmed that AR‐dependent activation of the *PSA* enhancer and promoter mediated by pS81 was coupled with activation of Pol II and the pTEFb complex. Mechanistically, we determined that CDK1 and CDK9 sustained the pS81 AR modification in the soluble and chromatin‐bound fractions of PCa cells, respectively. Finally, we demonstrated that CDK1 activity was maintained throughout the cell cycle, and that CDK1 inhibitors restored androgen sensitivity in CRPC tumor cells. Based on these findings, CDK1 and CDK9 could be targeted as pS81 kinases in patients with CRPC, either alone or in conjunction with direct AR antagonists.

## Graphical abstract

Androgen receptor (AR) is phosphorylated at Ser81 (pS81) in prostate cancer. By using chromatin immunoprecipitation analyses (ChIP, ChIP‐seq, reChIP and 3C‐ChIP), we demonstrated that pS81 is coupled to AR transactivation and enriched at AR‐activated enhancer‐promoter loops. pS81 is co‐stimulated by CDK1 and CDK9, both representing potential therapeutic targets in castration‐resistant prostate cancer patients, either alone or in conjunction with direct AR antagonists.

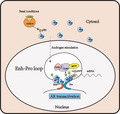

**Abbreviations**
3C: chromosome conformation capture
ADT: androgen deprivation therapies
AR: androgen receptor
ATAC‐Seq: assay for targeting accessible‐chromatin with high‐throughput sequencing
BETA: binding and expression target analysis
BRD4: bromodomain‐containing protein 4
CDK1: cyclin‐dependent kinases 1
CDK9: cyclin‐dependent kinases 9
CDS: steroid‐depleted
ChIP‐seq: chromatin immunoprecipitation analysis‐sequencing
CRPC: castration‐resistant prostate cancer
ddPCR: droplet digital PCR
DHT: dihydrotestosterone
DMEM: Dulbecco's modified Eagle's medium
Enh: enhancer
FACS: fluorescence‐activated cell sorting
IHC: immunohistochemistry
PCa: prostate cancer
PCR: polymerase chain reaction
Pro: promoter
pS81: AR serine 81 phosphorylation
PSA: prostate‐specific antigen
pTEFb: positive transcription elongation factor b
RNA Pol II: RNA polymerase II

## 1 Introduction

Androgen receptor (AR) is an essential molecule in prostate cancer (PCa) development and the major target in PCa therapy. In PCa cells, AR binds to thousands of genomic loci and regulates hundreds of gene promoters by recruiting transcription co‐factors that elicit chromatin remodeling and transcriptional activation [1, 2]. Most PCa patients respond to androgen deprivation therapies (ADT) but they generally relapse within a few years with castration‐resistant prostate cancer (CRPC). AR is still highly expressed in CRPC cells and many CRPC tumors respond to second‐line anti‐androgen agents, such as enzalutamide and the CYP17A1 inhibitor abiraterone. However, these responses are limited and mechanisms and targets underlying AR re‐activation in advanced CRPC remain to be established [3, 4, 5].

In response to androgen, AR undergoes phosphorylation at multiple proline‐directed sites [6, 7, 8]. The AR serine 81 (S81) is a proline‐directed phosphorylation residue that bears unusual features: it is embedded within a long poly‐glutamine (Poly‐Q) stretch with surface‐accessibility and it is the most androgen‐responsive phosphorylated site on AR [6, 9, 10, 11, 12]. Unlike phosphorylation at other AR residues, S81 phosphorylation (pS81) occurs over a prolonged time course that is correlated with AR‐target gene induction, indicating a mechanistic connection between pS81 and AR transactivation [9, 10, 13, 14, 15]. The expression of pS81 is also androgen dose‐dependently linked to AR transactivation [11]. Consistent with these observations, we previously reported that pS81 may function in AR nuclear localization and chromatin binding [9], and a recent study found that pS81 can mediate AR interaction with coactivator protein p300 [16]. In addition, the proline‐directed kinases CDK1 and CDK9 were identified to interact with AR and mediate S81 phosphorylation [6, 14, 17]. Canonically, CDK9 together with cyclin T forms the pTEFb complex that phosphorylates RNA polymerase II (RNA pol‐2) to elicit transcriptional elongation [18]. It has been proposed that CDK9 may similarly phosphorylate AR at S81 upon occupancy of the target gene promoter [17], providing a coupling mechanism where pS81 forms a complex with the pTEFb that simultaneously activates the RNA Pol II and transcription. Indeed, a recent report showed that AR can be reactivated through CDK9‐mediated phosphorylation, and co‐targeting CDK9 and AR can be an AR‐directed strategy in PCa [19].

CDK1 overexpression has been frequently observed in CRPC with increased G2‐M spectrum in the cell cycle [6, 20, 21, 22, 23]. In CRPC cells, hyperactivated CDK1 can mediate phosphorylation of S81 to activate AR in the absence of ligands or in response to residual androgens. Here we employed multiple approaches to assess a linkage of pS81 with AR transactivation at the chromatin loci of a subset of androgen‐stimulated genes. Using ChIP, ReChIP and 3C‐ChIP assay, we demonstrated an enrichment of pS81 at the AR‐activated enhancer‐promoter loop, where phosphorylated AR and activated RNA Pol II would share CDK9 for transactivation. Notably, we also found CDK1 and CDK9 co‐sustain pS81 expression across all cellular fractions, indicating pS81 is constantly equilibrated between cytoplasm and chromatin. In addition, amplified CDK1 can function throughout the cell cycle in CRPC cells and its inhibition can re‐sensitize CRPC cells to androgen. Together, our findings define the chromatin roles for Ser81‐phosphorylated AR on its transactivation and further validate the potential of CDK1 and CDK9 as therapeutic targets in PCa.

## 2 Materials and methods

### 2.1 Materials

As for human samples, the experiments were undertaken with the understanding and written consent of each subject. The study methodologies conformed to the standards set by the Declaration of Helsinki and were approved by the local ethics committee [Committee on Clinical Investigation, the institutional review board (IRB) for the Beth Israel Deaconess Medical Center, Boston, MA, USA]. The sources for compounds: DHT and nocodazole (Sigma, St. Louis, MO, USA); R1881 (Cat. NLP005; PerkinElmer, Waltham, MA, USA); RO‐3306 (Cat. ALX‐270‐463‐M001; Alexis Biochemicals, Lausen, Basel‐Landschaft, CH); Cdk9 Inhibitor II (iII, Cat. 238811; EMD Millipore, Billerica, MA, USA). The sources for the antibodies and control IgG: AR (N20, Cat. sc‐816; Santa Cruz, Dallas, TX, USA); pS81 (pAR‐Ser81, Cat. 07‐1375; EMD Millipore); pS2 (pRNA Pol II Ser2, ab193468; Abcam, Cambridge, MA, USA); pS5 (pRNA Pol II Ser5, ab5131; Abcam); CDK1 (CDC2, Cat. 9112; Cell Signaling, Danvers, MA, USA); pCDK1‐T161 (Cat. 9114; Cell Signaling); Cyclin A (Cat. sc‐596; Santa Cruz); Cyclin B1 (H‐433, Cat. sc‐752; Santa Cruz); CDK9 (Cat. sc‐8338; Santa Cruz); BRD4 (Cat. A301‐985A; Bethyl, Montgomery, TX, USA); Cyclin T1 (Santa Cruz; Cat. sc‐10750); Histone 3 (H3, Cat. ab1791; Abcam); pH3‐Ser10 (Cat. 06‐570; EMD Millipore); FoxA1 (Cat. Ab23738; Abcam); β‐Tubulin (Cat. MAB3408; EMD Millipore); PSA (Cat. K92110R; Meridian Life Science, Memphis, TN, USA); GAPDH (Cat. Ab9485; Abcam); and normal rabbit IgG (Cat. sc‐2027; Santa Cruz).

### 2.2 Immunohistochemistry

The immunohistochemistry (IHC) test was performed using the VectorStain EliteABC kit (Cat.H‐6100; Vector Laboratories, Burlingame, CA, USA) and its procedures, as outlined in the following steps.

Step1: Deparaffinization: the slides were baked at 60 °C for 1 h, then processed in the following order: xylene → xylene → 100% ethanol → 95% ethanol → 80% ethanol → 70% ethanol → 50% ethanol, each for 3 min. The slides were then rinsed with tap water 2 × 5 min.

Step 2: Nonezymatic antigen retrieval and epitope recovery: the slides were boiled in a steam container with 1× Diva Decloaker buffer (Biocare Medical #DV2004LX, MX) for 30 min and cooled down naturally, and the slides then rinsed with tap water.

Step3: Blocking: the slides were kept in 10% H_2_O_2_ for 5 min to quench endogenous peroxidase activity, and the slides rinsed in tap water and then blocked in 1% BSA/PBS with serum for 45 min.

Step 4: Antibody and avidin/biotin incubation: the antibodies were prepared in 1% BSA/PBS; for both the anti‐pS81 and anti‐AR antibody, the optimized final concentration was 100 ng·mL^−1^. The slides were incubated with the primary antibody overnight at 4 °C. After washing the slides in the PBS‐T buffer (0.05% Tween20) for 3 × 5 min, the slides were incubated with the biotin‐linked secondary antibody (1 : 400) in the blocking buffer (1% BSA/PBS with serum) at room temperature for 30 min. After washing the slides in the PBS‐T buffer for 3 × 5 min, the slides were incubated with the ABC reagent for 45 min, followed by washing in the PBS‐T buffer for 3 × 5 min.

Step 5: Chromagen detection and counterstain: the slides were developed using the DAB kit (Cat. SK‐4100; Vector Laboratories) and observed frequently under microscope for up to 5–20 min until the desired stains reached sufficient intensity; the slides were then immersed in tap water to stop the reaction. Upon washing the slides with running tap water, counter‐staining was conducted with 10% hematoxylin (Sigma MHS32‐1L) solution for 2 min. The slides were then rinsed with tap water.

Step 6: Dehydration and mounting: the slides were processed sequentially in 50% ethanol → 70% ethanol → 80% ethanol → 95% ethanol → 100% ethanol → xylene → xylene, each for 3 min, followed by mounting the slides with the non‐aqueous mounting reagent (PERMASLIP; Alban Scientific, St. Louis, MO, USA).

### 2.3 Bioinformatics and datasets

Time‐dependent androgen‐induction of AR‐regulated gene expression was based on LNCaP Affymetrix microarray (DHT, 10 nm; treatment for 2, 4, 8 and 24 h) [24]. GSEA study was performed as reported [25] with the following datasets: GSE32269; GSE11428, GSE31410 and GSE32356 [20, 22, 23]. For binding and expression target analysis (BETA) see http://cistrome.org/BETA/ [26]. AR binding in LNCaP cells was based on AR ChIP‐Seq (R1881 treated for 16 h; GSE14092) [27]. ChIP‐Seq annotation was performed using the WASHU Browser. The promoter peaks based on ± 3 kb of gene TSSs were identified using the R package ‘ChIPseeker’. The enhancer (roughly between 3 and 100 kb) was assigned to the nearest gene. Pair‐end sequencing reads from the AR ChIP‐seq experiment were aligned to the NBCI Build hg19 of the human genome with bowtie v2.3.5 [28] with default parameters. Only reads with a mapping quality > q5 were retained. Peak calling was performed on datasets with macs v2.2.6 software [29] using parameters ‘‐‐keep‐dup = 1’ and ‘‐‐SPMR’. According to the descending order of peaks of the *P*‐value, the top 5000 peaks of enhancer and promoter were identified, and then analyzed for *de novo* motif discovery in ChIP‐seq using HOMER [30]. Resultant bedGraph files were converted to big wiggle files using the University of California, Santa Cruz (UCSC) bedGraphToBigWig tool. Genomic signal within 2 kb of peaks was visualized using deepTools.

### 2.4 ChIP (chromatin immunoprecipitation), ChIP‐Seq, Re‐ChIP and 3C‐ChIP

#### 2.4.1 ChIP

The ChIP test was carried out as reported previously [17], and as briefly described here. For crosslinking: the cells in media were fixed by adding formaldehyde (1%, final concentration) and incubation at room temperature for 10 min, followed by quenching with glycine at a final concentration of 0.2 m. The cells were washed twice with pre‐chilled PBS, and then harvested in PBS. The nuclear fraction was extracted by first re‐suspending the pellet in 10 mL of LB1 buffer (50 mm Hepes‐KOH, pH 7.5; 140 mm NaCl; 1 mm EDTA; 10% glycerol; 0.5% NP‐40; 0.25% Triton X‐100) for 10 min at 4 °C. Cells were pelleted, re‐suspended in 10 mL of LB2 buffer (10 mm Tris‐HCl, pH 8.0; 200 mm NaCl; 1 mm EDTA; 0.5 mm EGTA) and mixed at 4 °C for 5 min. Cells were then pelleted and re‐suspended in 300 µL of LB3 buffer (10 mm Tris‐HCl, pH 8.0; 100 mm NaCl; 1 mm EDTA; 0.5 mm EGTA; 0.1% Na‐deoxycholate; 0.5% *N*‐lauroylsarcosine) and sonicated with a waterbath bioruptor (Diagenode, Denville, NJ, USA). Then 30 µL of 10% Triton X‐100 was added and the lysate was centrifuged for 10 min at 20 000 ***g*** to separate debris. The supernatant was then subjected to pre‐clearing and ChIP analysis, as described [17].

#### 2.4.2 ChIP‐seq

The ChIP step was carried out similar to Section 2.4.1 and the DNA was submitted for deep‐seq on the Illumina platform. In all, two AR and three pS81 ChIP samples were submitted for deep‐seq at the Molecular Biology Core Facilities at the Dana Farber Cancer Institute (MBCF at DFCI, Boston, MA, USA). All five ChIP‐Seq samples were based on independent tests of LNCaP cells in androgen‐depleted CDS medium. The DHT (10 nm) treatment conditions are: A1 (AR, DHT for 4 h); A2 (AR, DHT for 8 h); S1 (pS81, DHT for 4 h); S2 and S3 (pS81, DHT for 8 h). The ChIP‐Seq data were deposited in the GEO datasets (GSE166192).

#### 2.4.3 Re‐ChIP

The ReChIP test was carried out as reported previously [31].

#### 2.4.4 3C‐ChIP

##### Crosslinking

Formaldehyde was added to the cell culture medium for fixation at a final concentration of 1% (v/v). Incubation took place at room temperature for 10 min under gentle rotation. Glycine solution was added to a final concentration of 0.125 m to quench the reaction. Incubation was at room temperature for 5 min on rocker. After washing 2× in PBS, ~ 5 mL PBS were added and cells harvested by scratching. The medium was centrifuged for 5 min at 300 ***g*** at 4 °C and the supernatant discarded. Cell lysis was then conducted or the cells stored as flash‐freeze cell pellets in liquid nitrogen or dry ice/ethanol and at −80 °C.

##### Lysis and restriction digest

For each sample, 1 mL of ice‐cold cell lysis buffer (10 mm Tris‐HCl pH8.0, 10 mm NaCl, 0.2% NP40) was combined fresh with 1× protease inhibitors. Prepared cell lysis buffer 1 mL was added to each crosslinked cell pellet. The pellets were mixed in a vertex and the cell suspension incubated on ice for 20 min. After centrifugation at 2500 ***g*** for 5 min at 4 °C, the supernatant was discarded. The pelleted nuclei were washed once with 1 mL of ice‐cold cell lysis buffer. After centrifugation at 2500 ***g*** for 5 min at 4 °C, the supernatant was discarded. The pellets were gently re‐suspended in 100 μL of 0.5% SDS and incubated at 62 °C for 5–10 min. After heating was over, 290 μL of water and 50 μL of 10% Triton X‐100 (Sigma, 93443) were added to quench the SDS. Thorough mixing was carried out, avoiding excessive foaming. The suspension was incubated at 37 °C for 15 min. Finally, 50 μL of 10× REB (3C Restriction Enzyme Buffer) and 500 U of 3C restriction enzyme (*Pst*I, #R0140S; NEB, Ipswich, MA, USA) were added and chromatin digested overnight at 37 °C with rotation.

##### Proximity ligation and crosslink reversal

To inactivate 3C restriction enzyme, incubation at 80 °C for 20 min and then cooling naturally to room temperature was carried out. A 700‐μL aliquot of the ligation master mix below was added to each sample: 499 μL of water, 120 μL of 10× NEB T4 DNA ligase buffer (NEB, B0202), 70 μL of 10% Triton X‐100, 6 μL of 20 mg·mL^−1^ bovine serum albumin (NEB, B9000S), 5 μL of 400 U·μL^−1^ T4 DNA Ligase (NEB, M0202). Each sample (1.2 mL) was mixed by inverting, and split into two tubes, each containing 600 μL of mixture. Incubation at room temperature was carried out for 4 h or overnight with slow rotation. Finally, centrifugation at 2500 ***g*** was carried out for 5 min, and the supernatant removed.

##### CHIP

The pellets were re‐suspended in ChIP lysis buffer, followed by sonication, and the ChIP protocol was continued.

### 2.5 PCR, nest‐PCR, ddPCR and RT‐qPCR

The experimental data were analyzed by prism 7.0 (Graphpad, San Diego, CA, USA), presented as mean ± SD. Two‐tailed unpaired Student's *t*‐test was performed to calculate the statistical significance of two independent groups, whereas the analysis of variance (ANOVA) test was used in multiple testing. *P* < 0.05 was considered statistically significant: * *P* < 0.05, ** *P* < 0.01, *** *P* < 0.001.

#### 2.5.1 PCR and nest‐PCR

PCR round‐1 and round‐2 (nest‐PCR) were performed using Phusion Hot Start Flex DNA Polymerase (NEB, M0535L). The PCR condition for PCR round 1 was: 98 °C for 2 min; 60× (98 °C for 15 s; 63 °C for 30 s; 72 °C for 30 s); 72 °C for 7 min. The PCR condition for PCR round 2 was : 98 °C for 2 min; 60× (98 °C for 15 s; 63 °C for 30 s; 72 °C for 20 s); 72 °C for 7 min.

#### 2.5.2 Droplet digital PCR

The droplet digital PCR (ddPCR) was performed on the Bio‐Rad QX200 AutoDG Droplet Digital PCR System (Bio‐Rad, Hercules, CA, USA) that uses a C1000 Touch Thermal Cycler with 96–Deep Well Reaction Module. The ddPCR Probe no UNG cycling conditions: 95 °C/10 min; 40 cycles of 94 °C/30 s and 60 °C/1 min; 98 °C/10 min.

#### 2.5.3 RT‐qPCR

The qPCR analysis was performed with the SYBR Green method on the Step‐One‐Plus Real‐time PCR system (Cat. 4309155; Applied Biosystems, Foster, CA, USA), with the following primers:

**Table:** 

PSA enhancer:	Forward, 5′‐GCCTGGATCTGAGAGAGATATCATC‐3′
	Reverse, 5′‐ACACCTTTTTTTTTCTGGATTGTTG‐3′
NKX3‐1‐ARE:	Forward, 5′‐CTGGCAAAGAGCATCTAGGG‐3′
	Reverse, 5′‐GGCACTTCCTGAGCAAACTT‐3′
ATAD2‐ARE:	Forward, 5′‐AGCATGTGTTTGCATGGGTA‐3′
	Reverse, 5′‐CACAGGGAAAGATCACTAAGACC‐3′

Additional primer/probe information is provided in Fig. S4.

### 2.6 RNA isolation and real‐time RT‐PCR analysis

Total RNA was isolated with the TriZOL reagent (Ambion, Austin, TX, USA). Real‐time RT‐PCR analysis was carried out with the TaqMan One‐Step RT‐PCR Master Mix Reagents (Cat. 4309169; Applied Biosystems). The TaqMan primer‐probe sets for KLK3/PSA (FAM labeled, Cat. PN4351370), NKX3‐1 (FAM labeled, Cat. Hs00171834_m1), ATAD2 (FAM labeled, Cat. Hs00204205_m1) and the internal control GAPDH (VIC‐TAMRA labeled, Cat. 4310884E) transcripts were from Applied Biosystems.

### 2.7 FACS analysis

Hoechst 33342 staining and FACS analysis are described below: LNCaP cells were grown in RPMI medium containing 5% CDS or 10% FBS for 2 days as indicated, and LNCaP‐Abl cells were grown in phenol red‐free RPMI medium containing 10% CDS for 2 days, followed by treatment without or with 10 nm of DHT for 24 h as indicated. Hoechst 33342 (at a final concentration of 4 μg·mL^−1^; Sigma) was added directly to the medium and the cells were further incubated for 60 min at 37 °C. The cells were then trypsinized and subjected to FACS analysis using the Beckman Coulter MoFlo Astrios (Brea, CA, USA). Cells gated at 2n and 4n were sorted as G0/G1 and G2/M populations, respectively. The sorted cells were then boiled in 2% SDS, normalized and subjected to blotting. PI staining and FACS analysis were carried out as described previously [9].

### 2.8 Cytoplasmic, nuclear and chromatin fractionation

The assay was carried out with the Subcellular Protein Fractionation Kit (Cat. 78840; Pierce, Rockford, IL, USA), following the manufacturer's directions. With this kit it is possible to assess cytoplasmic, membrane, soluble nuclear, chromatin‐bound and cytoskeletal protein fractions. In this report, we focused on the cytoplasmic, soluble nuclear and chromatin‐bound fractions, with the insoluble chromatin‐bound fraction being released by micrococcal nuclease (MNase).

### 2.9 Cell culture and transfection

All cell lines were from ATCC (Manassas, VA, USA). The LNCaP**‐**Abl cell line was maintained in phenol‐red‐free RPMI‐1640 medium (Cat. 118035‐030; Gibco, Grand Island, NY, USA) containing 10% CDS. LNCaP were grown in the RPMI‐1640 containing 10% FBS; C4‐2 and Cos1 cells were grown in Dulbecco's modified Eagle's medium (DMEM) containing 10% FBS; and 293T cells were grown in DMEM with 5% FBS. For androgen‐starving conditions, cells were grown in medium containing 5% CDS. Plasmid transfection was carried out using Lipofectamine 2000 (Cat. P/N52887; Invitrogen, Carlsbad, CA, USA).

### 2.10 Cell proliferation

Cells were cultured in 96‐well plates for 2 days and then treated for 3 days as indicated. The cell counting analysis was performed with the CellTiter‐Glo assay kit (Cat. G7571; Promega, Madison, WI, USA), following the manufacturer's manual.

### 2.11 Animal xenografts

Six‐ to 8‐week‐old male ICR/scid mice (IcrTac:ICR‐Prkdc<scid>) from Taconic (Taconic Biosciences, Inc., Germantown, NY, USA) were used to generate PCa xenografts. The mice are housed in Allentown HEPA‐filtered, individually ventilated cages with ducted HEPA exhaust and automatic watering. Irradiated food and hyperchlorinated, reverse osmosis water are provided to all cages. Rodent racks, cages and bedding are autoclaved in double‐door bulk autoclaves prior to use. The mice were injected subcutaneously with 2 million VCaP/LAPC4 cells in 50% Matrigel. When the number of xenografts reached ~ 1000 mm^3^, biopsies were obtained and the mice were castrated [32]. Additional biopsies were obtained 4 days after castration, and the tumors were harvested at relapse. All animal experiments were approved by the Beth Israel Deaconess Medical Center (BIDMC) Institutional Animal Care and Use Committee (IACUC) and were performed in accordance with institutional and national guidelines.

## 3 Results

### 3.1 Nuclear expression of S81‐phosphorylated AR in metastatic PCa rapid biopsy and in PCa xenografts is correlated with AR transactivation

**Fig. 1:**
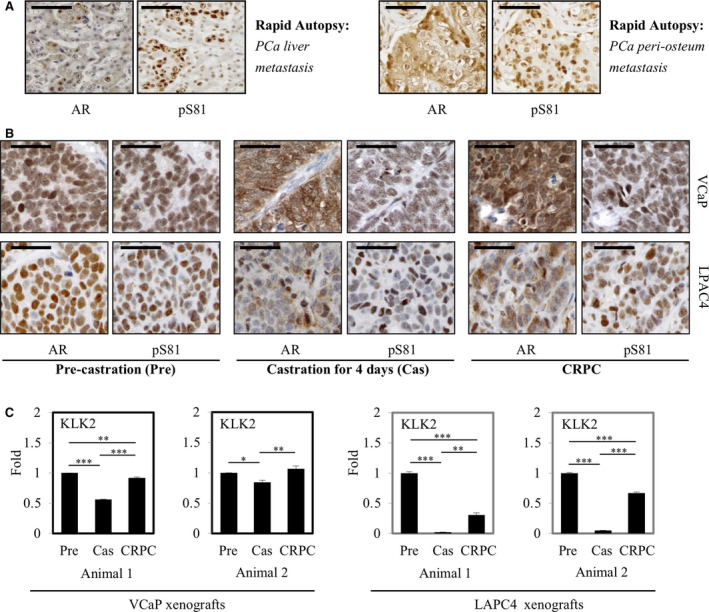
Nuclear expression of Ser81‐phosphorylated AR (pS81) in metastatic PCa rapid biopsy and in PCa xenografts is correlated with AR transactivation. (A) AR and pS81 staining based on rapid autopsied PCa samples collected from indicated metastatic sites of a PCa patient having received anti‐androgen treatment. Scale bars: 25 μm. (B) AR and pS81 staining of VCaP (upper panels) and LAPC4 (lower panels) xenografts through castration and progression to CRPC. Pre: pre‐castration; Cas: castration for 4 days; CRPC: recurrent CRPC [32]. Scale bars: 25 μm. (C) Real‐time RT‐PCR analysis of KLK2 expression in representative mice bearing VCaP and LAPC4 xenografts, respectively. Data are presented as mean ± SD. Statistical significance was determined by one‐way ANOVA; *P* < 0.05 was considered statistically significant: * *P* < 0.05, ** *P* < 0.01, *** *P* < 0.001.

In previous reports, we used an affinity‐purified polyclonal phospho‐AR‐Ser81 (pS81) antibody that was validated by peptide‐competition assay in both western blotting and an optimized IHC staining protocol [11, 17]. In the end, we stained pS81 on multiple clinical PCa samples, including benign, primary PCa, CRPC and metastasis, and VCaP xenografts before and after castration [11]. In this report, we similarly stained rapid biopsy samples from a patient who had undergone various anti‐androgen treatments. As shown (Fig. 1A), IHC tests in the liver and bone metastases all indicated that pS81 has a specific and intense nuclear distribution pattern, despite diffused staining of total AR in both the cytoplasm and nucleus. Overall, these observations substantiated our previous findings of an enrichment of pS81 in the metastatic CRPC nucleus.

We next examined total AR and pS81 by IHC in castration‐sensitive and castration‐resistant VCaP and LAPC4 xenografts. In the pre‐castration xenografts, the AR and pS81 antibodies showed staining in both the cytoplasm and nucleus, with more preferential accumulation of the pS81‐AR in the nucleus (Fig. 1B). In xenografts harvested 4 days after castration there was a shift towards cytoplasmic distribution for total AR, with fewer cells showing nuclear pS81; those that did show pS81 staining, demonstrated decreased intensity, consistent with the decrease in androgen. In relapsed xenografts (about 6 weeks after castration), restoration of AR activity was likely driven by mechanisms including increased AR and intratumoral androgen synthesis. Significantly, total AR in these xenografts was highly expressed in the cytoplasm and nucleus, and pS81 was highly expressed and predominantly nuclear (Fig. 1B). These findings are consistent with our previous reports of nuclear pS81 distribution in CPRC xenografts and clinical samples [11].

Of importance, in these xenografts there is a consistent alignment of pS81 nuclear expression and AR‐activated gene expression profiling, as assessed by monitoring the expression of KLK2 messages in both xenograft models (Fig. 1C). Tests on additional AR signature genes had similar results [32]. These xenografts studies support a functional alignment between nuclear pS81 and AR‐mediated transcription *in vivo*.

### 3.2 S81 phosphorylation on chromatin is intrinsically linked to AR‐mediated transactivation

**Fig. 2:**
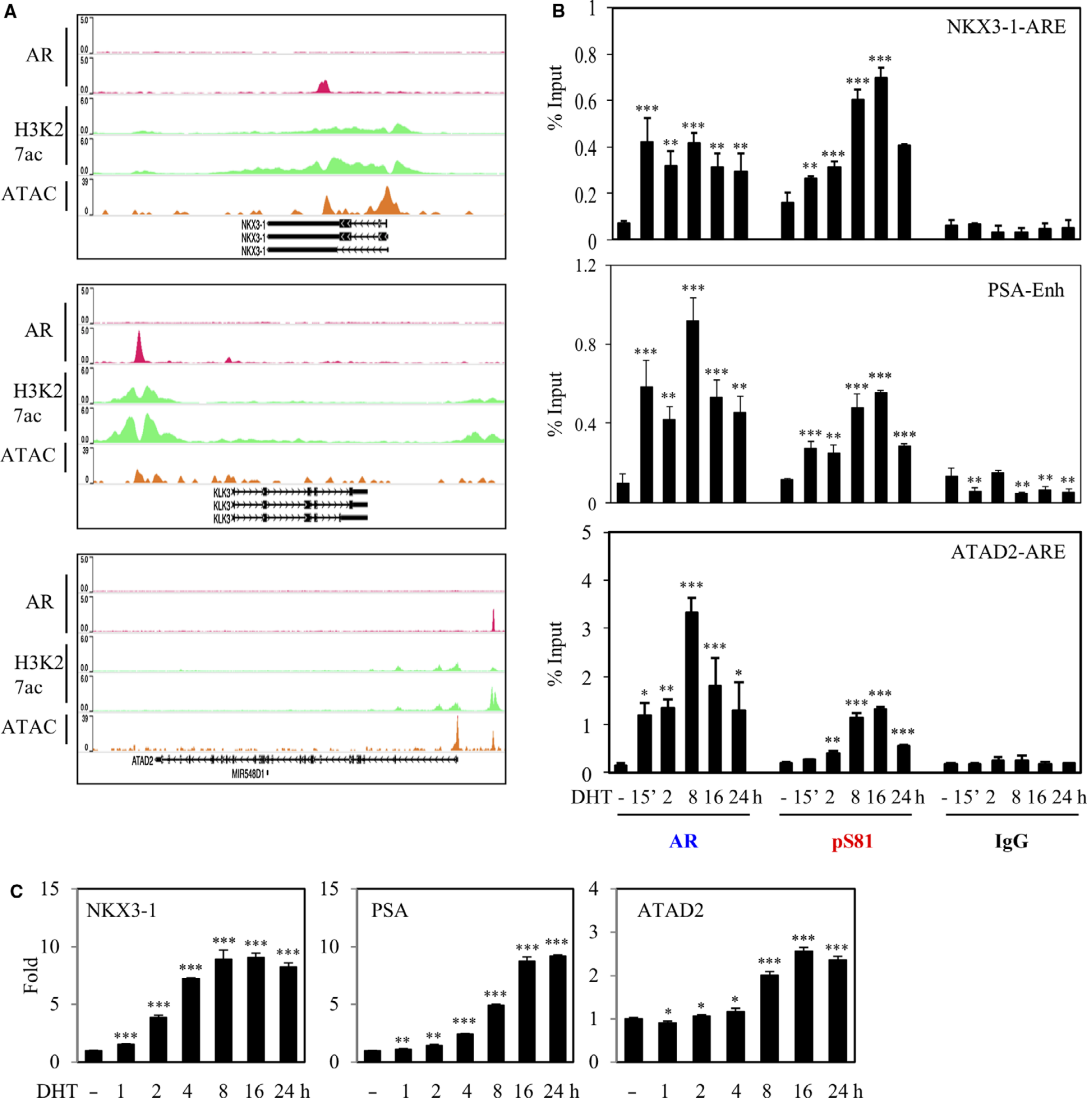
pS81 chromatin expression is intrinsically linked to AR‐mediated transactivation. (A) Annotation of LNCaP AR ChIP‐seq (−/+ androgen, GSM3138622 and GSM3138626, respectively), H3K27Ac ChIP‐seq (−/+ androgen, GSM1249447) and ATAC‐Seq (GSM4130898) in the loci of three representative androgen‐responsive genes: early‐induced gene NKX3‐1, intermediate‐induced gene PSA, and late‐induced gene ATAD2. The alignment was based on WASHU Brower. (B) LNCaP cells in androgen‐depleted medium were stimulated for indicated time‐points with 10 nm of DHT and then harvested for ChIP analysis of AR and pS81, with IgG as negative control. AR binding sites, as the major peaks in (A), were subjected to ChIP‐qPCR analyses. (C) LNCaP cells in androgen‐depleted medium were stimulated for indicated time‐points with 10 nm of DHT and then total RNA was harvested for real‐time RT‐PCR analysis. The ratio (fold) was calculated based on the value of specific gene expression as normalized to the internal control GAPDH, with the un‐liganded control set to 1. Data are presented as mean ± SD of three biologically independent replicates. Statistical significance was calculated by one‐way ANOVA; *P* < 0.05 was considered statistically significant: * *P* < 0.05, ** *P* < 0.01, *** *P* < 0.001.

In the classical androgen‐sensitive PCa LNCaP cells, pS81 expression is induced by androgen in a time‐ and dose‐dependent manner and its induction correlates with AR‐mediated gene stimulation, indicating a mechanistic connection between pS81 and AR transactivation [6, 11]. To examine specifically the functions of pS81 on chromatin, we performed ChIP assays to test the hypothesis that the time course for activating distinct AR‐mediated genes is in alignment with locus‐specific pS81 occupancy. For this purpose, we selected an early androgen‐responsive gene (*NKX3.1*), an intermediate androgen‐responsive gene (*KLK3/PSA*) and a late androgen‐responsive gene (*ATAD2*). The grouping was based on an analysis of time‐dependent gene stimulation in LNCaP Affymetrix microarray in conjunction with AR binding based on LNCaP AR ChIP‐Seq datasets (Figs S1 and S2). Interestingly, the gene loci of early androgen‐responsive gene *NKX3‐1* and intermediate androgen‐responsive gene *PSA* have strong AR binding that is associated with open chromatin as marked by pronounced locus‐specific H3K27Ac expression and ATAC‐Seq signal. In contrast, the late androgen‐responsive gene *ATAD2* gene loci has relative weak AR binding, and the AR binding sites are marked by less pronounced H3K27ac binding and ATAC‐Seq signal, indicating less chromatin accessibility (Fig. 2A).

ChIP‐qPCR studies using total AR and pS81‐specific antibody demonstrated that androgen quickly induced strong pS81 binding at the NKX3‐1 and PSA AR enhancers, whereas pS81 chromatin distribution was weak at the *ATAD2* gene loci and was stimulated at a delayed time course (Fig. 2B). Furthermore, the time course of chromatin pS81 expression was generally in line with induction of specific gene messages (Figs 2C, S1 and S2). These findings support an association between chromatin openness and pS81 binding, and solidify evidence for a mechanistic connection between chromatin‐bound pS81 and AR‐mediated transactivation.

### 3.3 pS81 expression is highly enriched in the AR‐activated gene promoter

**Fig. 3:**
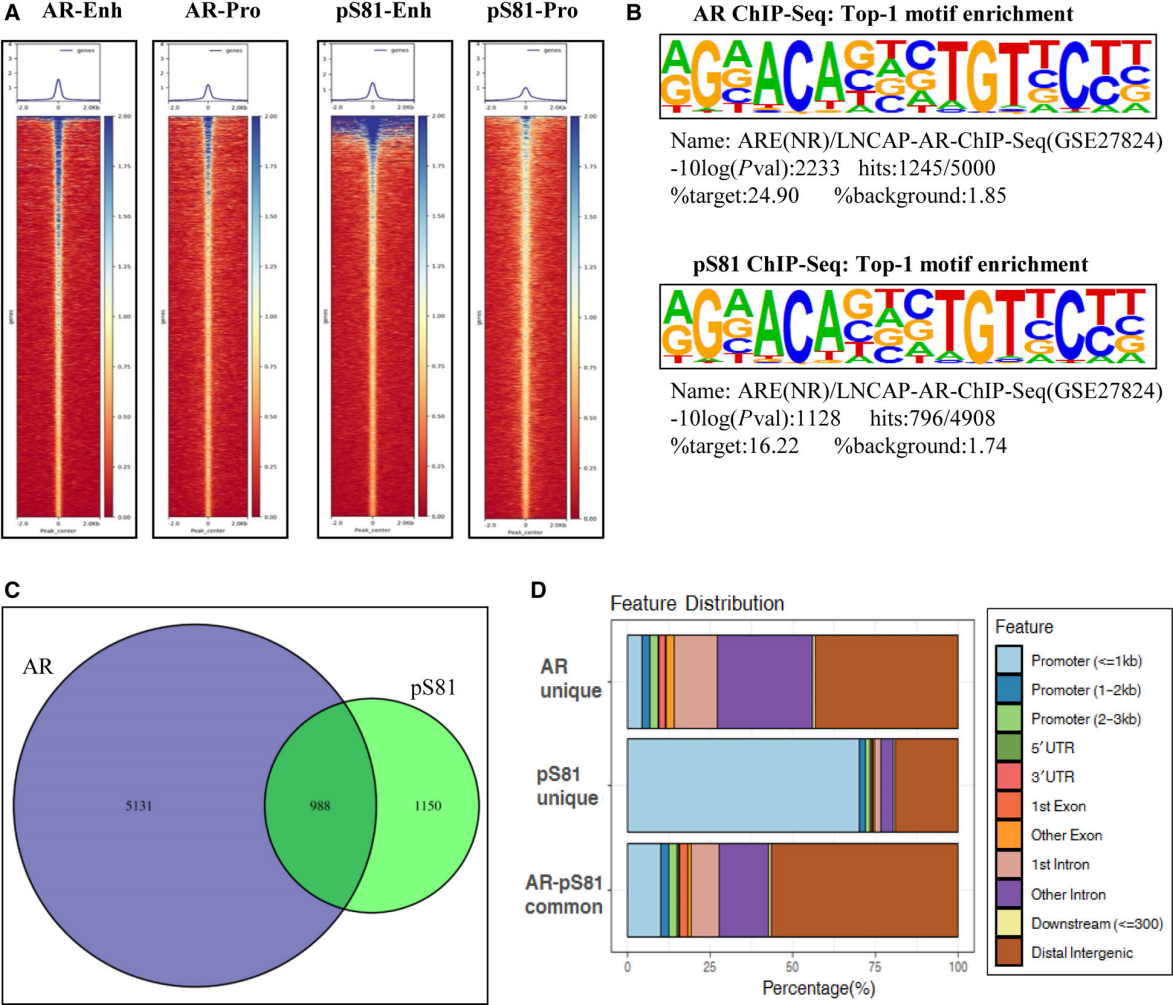
AR and pS81 ChIP‐seq analyses indicated they had both common and distinct binding profiles and pS81 was enriched on AR‐activated promoter. LNCaP cells in androgen‐depleted medium were stimulated with 10 nm of DHT for 8 h and then harvested for ChIP‐seq analysis using Illumina platform. (A) Heatmap analyses of global AR and pS81 enrichment at enhancer versus promoter regions along the genome. (B) Global motif enrichment analyses of AR and pS81 ChIP‐seq; in both cases AR motif was ranked as the Top‐1 hit. (C) Venn diagram of AR and pS81 ChIP‐seq. (D) Barplot using ChIPseeker to characterize AR and pS81 binding profiles along the genome.

As our findings identified a connection between pS81 and AR‐mediated gene expression, we next conducted global analysis of phosphorylated AR binding along the genome to define further the mechanistic link. For this purpose, we performed ChIP analyses in LNCaP cells stimulated by androgen, followed by deep‐seq on the Illumina platform. As exemplified in Fig. 3A, both AR and pS81 signals were globally centered on the enhancer and promoter regions. Motif analyses demonstrated that classic AR binding motif was the top annotation hit for both AR and pS81 ChIP‐Seq datasets (Fig. 3B), validating our analyses. AR is known to bind to distal enhancers, and next we aligned AR and pS81 global binding profiling. Interestingly, pS81 have ~ 54% unique sites and ~ 46% common sites with total AR (Fig. 3C), indicating that they share a distinct chromatin distribution profiling. Further analyses showed pS81 had higher enrichment at the promoter, whereas total AR was more enriched at the distal enhancer (Fig. 3D).

**Fig. 4:**
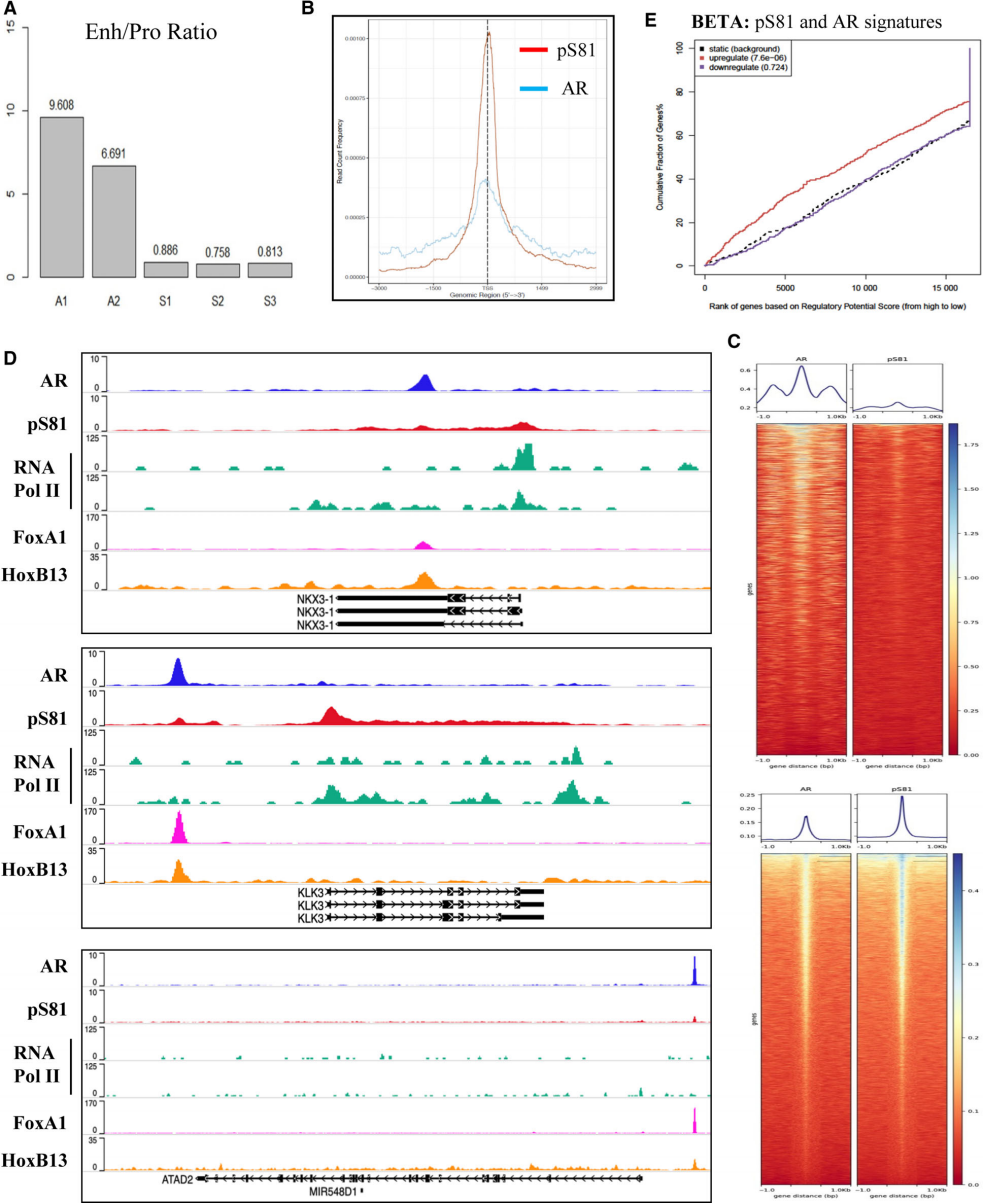
In AR‐activated genomic loci, Ser81‐phosphorylated AR is highly expressed in the vicinity of the enhancer and promoter. (A) Global calculation of pS81/AR ratio based on AR ChIP‐Seq and pS81 ChIP‐Seq datasets generated in this report. IN all, two AR (A1‐A2) and three pS81 (S1‐S3) ChIP samples were submitted for deep‐seq. These independent samples were all based on LNCaP in androgen‐depleted medium. DHT (10 nm) treatment conditions: A1 (AR, DHT for 4 h); A2 (AR, DHT for 8 h); S1 (pS81, DHT for 4 h); S2 and S3 (pS81, DHT for 8 h). (B) AR and pS81 peak binding plot at the transcriptional starting sites (TSS) indicated a robust enrichment of Ser81‐phosphorylated AR at the proximal promoter. (C) Global profiling of AR and pS81 on AR‐occupied enhancers (upper panels) vs. promoters (lower panels) were analyzed based on H3K27ac ChIP‐Seq, AR ChIP‐Seq and pS81 ChIP‐Seq datasets using deepTools. (D) Track alignment of LNCaP AR ChIP‐Seq (A2) and pS81 ChIP‐Seq (S2), RNA Pol II ChIP‐Seq (−/+ androgen, GSM353617 and GSM353618, respectively), FoxA1 ChIP‐Seq (GSM2480813) and HoxB13 ChIP‐Seq (GSM2480817) at the NKX3‐1, KLK3 and ATAD2 gene loci. The data range for both AR and pS81 signals was set at 0–10. The alignment was based on WASHU Browser. (E) BETA study of pS81 ChIP‐Seq and AR signature genes in LNCaP (GSE118152).

We reason that the discrepancy between AR and pS81 binding is due both to the stringency of the analyses and the fact that AR is enriched at distal enhancers, whereas pS81 has preferential enrichment at proximal promoters. Consistently, the two AR ChIP‐seq datasets (A1‐A2) yielded a much higher Enh/Pro ratio than that of the three pS81 datasets (S1–S3) (Figs 4A and S3). In comparison, pS81 had a more balanced distribution between enhancer and promoter (Fig. 4A–C and Fig. S3), an indication that AR is phosphorylated at Ser81 in the vicinity of the Enh‐Pro.

To specify further the AR and pS81 binding profiling, we next showed the tracks of AR, pS81 and RNA Pol II signals at AR‐activated gene loci, together with the pioneer factors FoxA1 and HoxB13 (Fig. 4D). At all three gene enhancers there was greater AR occupancy than pS81; in contrast, in the early AR‐stimulated gene promoters (NKX3‐1 and KLK3), there was greater enrichment of pS81 as compared with total AR, whereas in the late AR‐stimulated gene promoter (*ATAD2*), the pS81 and total AR signals were comparable. The overall pS81 and AR signals were higher in the early activated genes than in the late stimulated genes. Additionally, an alignment of pS81 with RNA Pol II also indicated their approximate distribution in the promoter and along the gene body (Fig. 4D). Of significance, binding and expression target analysis (BETA) that combines gene expression changes with genome‐wide binding data indicated that the pS81 binding profile was correlated with AR activated but not repressed signatures (Fig. 4E). These findings support an active engagement of Ser81‐phosphorylated AR with the Enh‐Pro looping complex mediating RNA Pol II activation [33].

### 3.4 3C‐ChIP analyses confirmed an enrichment of pS81 at the PSA gene enhancer and promoter looping complex

**Fig. 5:**
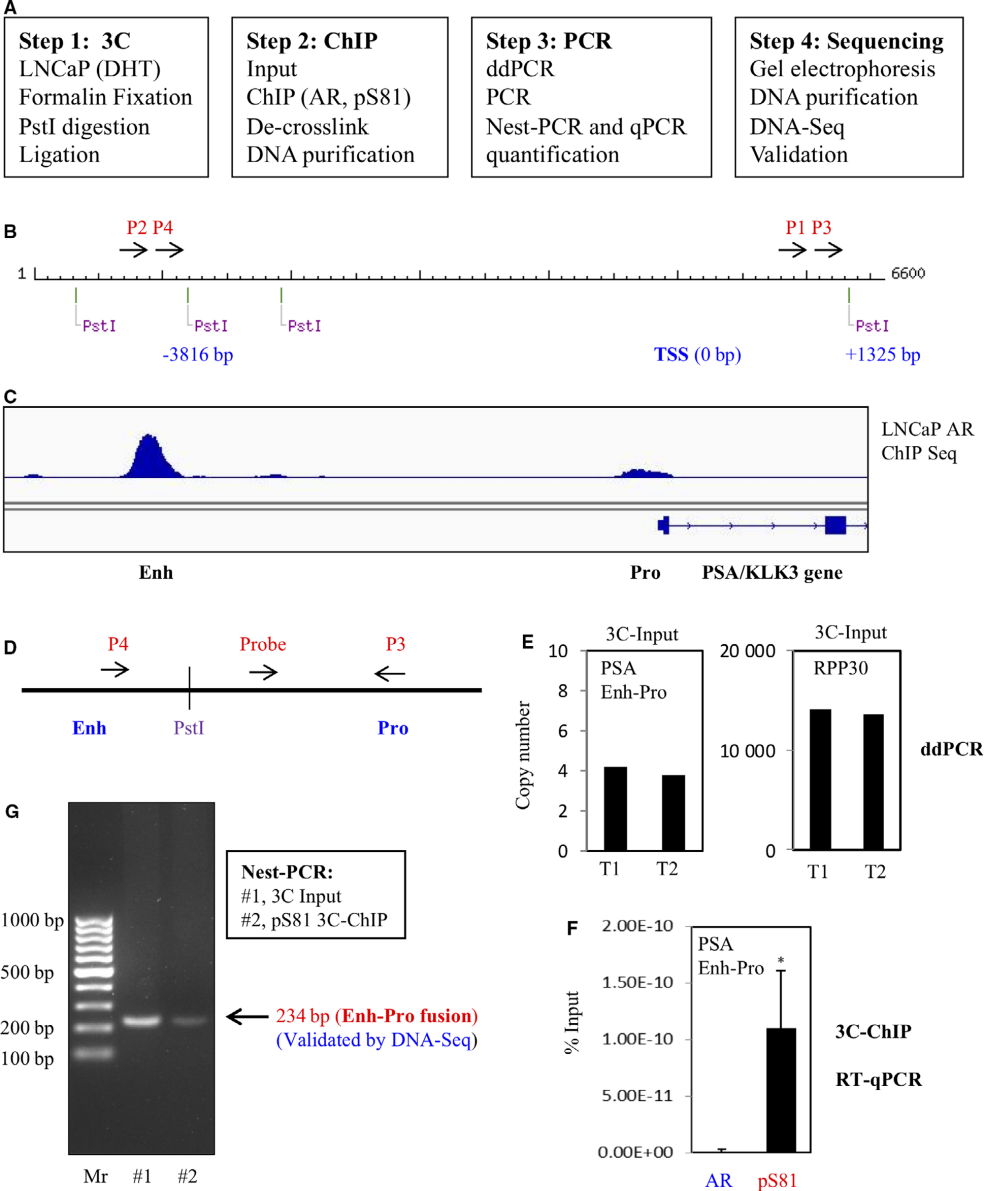
3C‐ChIP analyses demonstrated an enrichment of pS81 at the PSA gene enhancer and promoter looping complex. (A) Outline of the experimental plan. (B) Display of the PSA gene regulatory region (~ 6.6 kb) featured with *Pst*I sites, transcriptional starting site (TSS) and PCR primers. The linear distance between the targeted PSA Enh‐Pro *Pst*I sites is 5141 bp. (C) Alignment of the PSA gene locus based on AR ChIP‐Seq in LNCaP cells (GSE14092). (D,E) Droplet digital PCR (ddPCR) to assess the *Pst*I digestion, followed ligation efficiency in the 3C test. The targeted PSA Enh‐Pro hybrid was assessed with specific primers and a FAM probe with QSY as quencher (D). As shown, in two independent ddPCR reads (20 μL reaction each), the following copy numbers were recorded: 4.2 and 3.8 for the PSA Enh‐Pro amplicons vs. 14 120 and 13 640 for that reference RPP30 that does not contain *Pst*I in its amplicon, respectively (E). Two repeated tests: T1 (test 1) and T2 (test 2). (F) PCR round 1 products were used for SYBR Green real‐time (RT) qPCR to quantify the 3C‐ChIP products based on pS81 and total AR, respectively. Each test includes three biologically independent replicates and results are presented as mean ± SD. Two‐tailed unpaired Student's *t*‐test was performed to calculate the statistical significance. *P* < 0.05 was considered statistically significant, as marked by *. (G) Nest‐PCR identified a single specific 3C band in the input and pS81 ChIP product, with an expected size at 234 bp. A 100‐bp gene ruler was used as DNA marker (Mr, Marker, Thermo Fisher, SM0243). More background information is included in Figs S4 and S5.

To address specifically a role of pS81 in AR‐activated Enh‐Pro loops, we developed a 3C‐based ChIP test based on established 3C protocols [34, 35] followed by an adapted ChIP assay (Fig. 5A). The 3C step was based on *Pst*I digestion of the PSA regulatory region – two *Pst*I sites that are ~ 5 kb apart and in proximity to the enhancer and promoter, respectively (Figs 5B,C and S4). The distal arrangement of these two *Pst*I sites and the directional placement of the PCR primers would effectively discount amplification background. Next, we performed the 3C test in LNCaP cells under DHT treatment (10 nm for 12 h) and then applied the droplet digital PCR (ddPCR) system to assess the 3C digestion and ligation efficiency (Fig. 5D,E). The analyses were based on targeted amplification of the PSA Enh‐Pro hybrid, which was assessed with specific primers and a FAM probe together with the reference reaction that was based on the copy number gene *RPP30* with a HEX probe. Without considering the variations in the amplification efficiency resulted from different genomic loci and PCR primer/probe sets, two independent ddPCR readouts consistently indicated an efficacy of Enh‐Pro fusion at 0.0288% (± 0.0013), or 3200–3750 cells/event, roughly in the range of a typical 3C test, where locus‐specific ligation events occur in 1/2000 to 1/20 000 mammalian cells [34].

The above 3C test indicated that we had generated the PSA Enh‐Pro looping complex in androgen‐stimulated LNCaP cells. To determine whether Ser81‐phosphorylated AR was preferentially distributed in the loop, the 3C ligation mixtures were then subjected to ChIP analysis using pS81 and AR antibodies, respectively. Due to the low productivities in both 3C and ChIP assays, we next adapted two‐round nest‐PCR analyses with the first round PCR using the hot‐start PCR system, which is robust and and has a high fidelity in amplification (Figs 5B and S4). The PCR (round 1) products were assessed using SYBR Green real‐time (RT) qPCR, which showed a massive enrichment of the 3C products with the pS81 antibody over the total AR antibody (Fig. 5F). To verify the specificity of the amplification, we then used a nest‐PCR (round 2) to amplify the PCR (round 1) products similarly with the hot‐start PCR system. The specificities of all the above PCR reactions were also safeguarded by the uniqueness of the primers and probes used in this report, all of which were verified by blast against the human genome and transcripts (Fig. S4). Indeed, the nest‐PCR yielded a clean background and produced a single specific 3C band in both the input and the pS81 ChIP samples, which had a theoretical size of 234 bp (Fig. 5G). The PCR products were then purified and submitted for DNA sequencing, which confirmed that a PSA Enh‐Pro fusion occurred exactly at the targeted *Pst*I sites in both the input and the pS81 ChIP samples (Fig. S5).

### 3.5 pS81 expression on the chromatin is coupled to AR‐dependent transactivation and is co‐sustained by CDK1 and CDK9

**Fig. 6:**
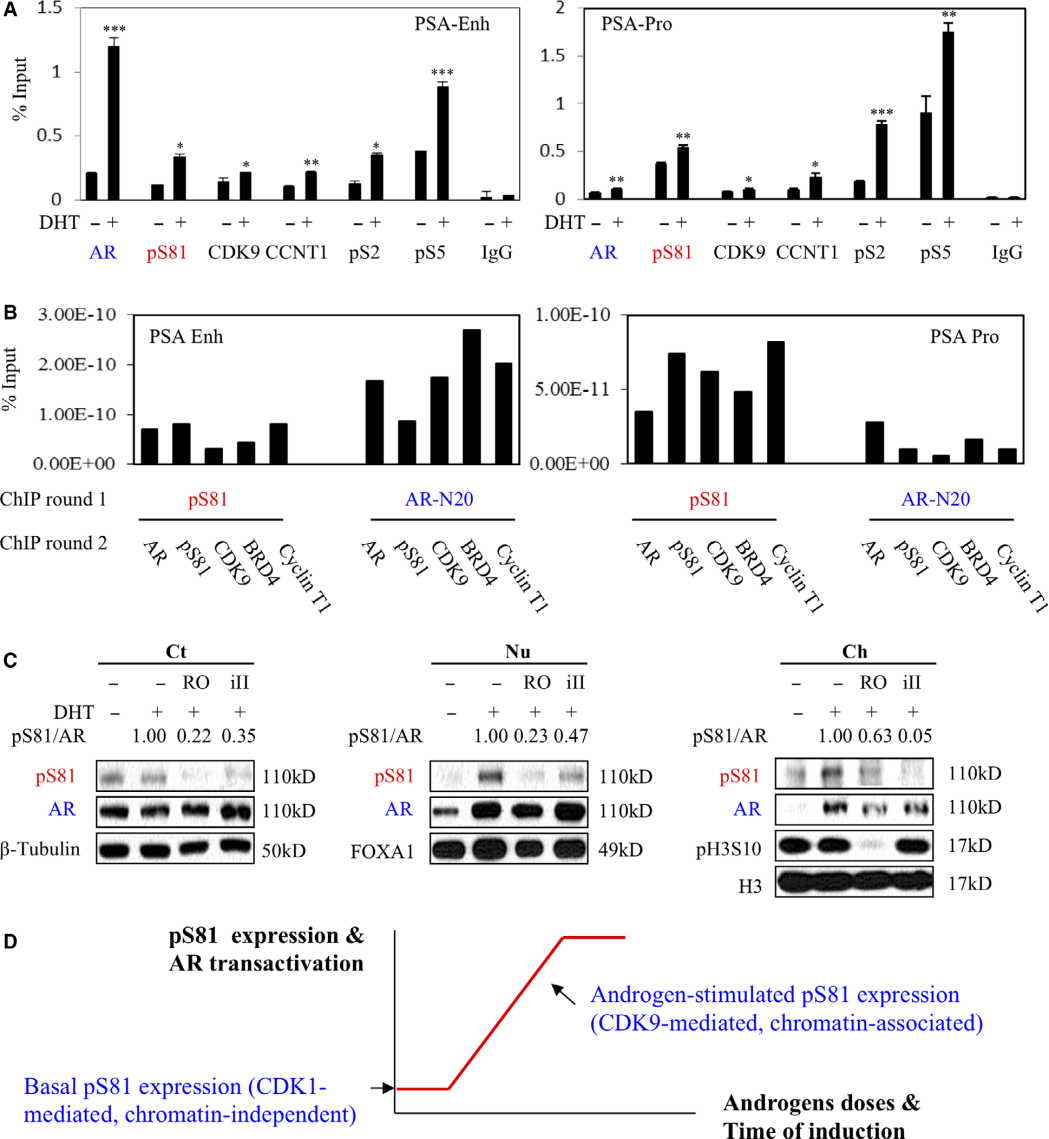
pS81 expression on the chromatin was coupled to AR‐dependent transactivation and co‐sustained by CDK1 and CDK9. (A) ChIP‐qPCR analyses of LNCaP cells in androgen‐depleted medium that were treated with DHT (10 nm, 24 h). CCNT1, cyclin T1; pS2, Ser2 phosphorylated RNA Pol II; pS5, Ser5‐phosphorylated RNA Pol II. (B) Re‐ChIP analysis of LNCaP cells in androgen‐depleted medium treated with 10 nm DHT for 24 h. The antibodies used for the first and second round of ChIP analysis are indicated; binding of specific factors to PSA enhancer and PSA‐promoter loci was monitored. (C) LNCaP cells in androgen‐depleted medium were pretreated for 2 h with CDK1 and CDK9 inhibitors, and 10 nm of DHT was then added for 15 min, followed by cellular fractionation. The insoluble chromatin‐bound fraction (Ch) was released using MNase. CDK1 inhibitor (RO: RO‐3306, 10 µm) and CDK9 inhibitor II (iII, 50 μm). Beta‐tubulin, FoxA1 and Histone H3 were used as markers for cytoplasmic, nuclear and chromatin‐bound proteins, respectively. Phospho‐H3‐Ser10 (pH3S10) was used as a marker for CDK1 activity that peaks in mitosis. pS81 and AR signals of the DHT pulsed samples were quantified using imagej (U. S. National Institutes of Health, Bethesda, Maryland, USA) and the normalized pS81/AR ratio is shown. (D) A model was proposed to outline pS81 activities that were co‐sustained by CDK1 acting under basal conditions and in soluble environments and by CDK9 working under androgen stimulation and chromatin‐dependence. The amplification of CDK1 in CRPC makes it a potential therapeutic target. Each test includes three biologically independent replicates and results are presented as mean ± SD. Statistical significance was determined by two‐tailed unpaired Student's *t*‐test; *P* < 0.05 was considered statistically significant: * *P* < 0.05, ** *P* < 0.01, *** *P* < 0.001.

Our previous reports indicated that CDK9 can stimulate S81 phosphorylation of AR in the presence of androgen, whereas pS81 expression was more dependent on CDK1 under androgen ablation [17]. Consistent with our findings, a recent study demonstrated that AR re‐activation can be achieved by CDK9‐mediated phosphorylation and that PCa can be co‐targeted by CDK9 inhibitor and AR antagonist [19]. Indeed, we determined by ChIP‐qPCR that the robust occupancy of pS81 (but not total AR) at the KLK3 promoter was correlated with the recruitment of pTEFb (CDK9/Cyclin) complex and transcriptional active RNA Pol II, as marked by pS2 and pS5 (Fig. 6A). Notably, the chromatin expression of pS81 was similar between PSA enhancer and promoter, further evidence of its distribution in the Enh‐Pro looping complex.

Next, to specify pS81 functions on the chromatin we assessed its engagement with the CDK9 complex. For this purpose, we performed re‐ChIP analysis, which is based on two rounds of ChIP assays that are sandwiched with an intermediate step to release the precipitation upon the completion of the first round ChIP test. As shown in Figs 6B and S6, re‐ChIP assays at the PSA Enh demonstrated that total AR had a higher enrichment than pS81 for the pTEFb/BRD4 complex, which is consistent with a higher total AR signal in the first round of ChIP assay on the enhancer (Figs 4B and 6A). Importantly, re‐ChIP assay at the PSA‐Pro demonstrated that enrichment of pS81 was higher than that of total AR for the pTEFb/BRD4 complex. These findings strongly support that pS81 is looped at AR‐activated Enh‐Pro loci, where it is complexed with the elongation complex (pTEFb/BRD4 and RNA Pol‐2).

As a complementary approach to verify the above findings further, we next carried out biochemical tests to similarly assess pS81 expression in distinct cellular compartments. For this purpose, we isolated soluble proteins from the cytoplasm and from the nucleus. The insoluble fraction was then subjected to MNase digestion to release the chromatin‐bound proteins. As shown in Fig. 6C, DHT quickly induced nuclear and chromatin localization of AR and pS81, with the nuclear and chromatin‐bound AR having a higher proportion of pS81 than cytoplasmic AR did. Significantly, both CDK1 and CDK9 inhibitors attenuated pS81 expression in all cellular chambers, with CDK1 inhibitor more effectively repressing pS81 in the soluble fractions and CDK9 inhibitor more severely reducing pS81 on the chromatin.

Together, we outlined these findings on pS81 in a graphic model (Fig. 6D). Under basal conditions CDK1 stimulates pS81 in the soluble cell compartments, independent of chromatin association. While under androgen‐stimulated conditions, CDK9 stimulates pS81 on the chromatin, and results in transcriptional activation. The pS81 repressive effects of both CDK1 and CDK9 inhibitors in all cellular chambers imply that AR undergoes dynamic re‐distribution and constant equilibration within the cellular spaces of cytoplasm, nucleus and chromatin.

### 3.6 CDK1 activity is required to activate AR‐dependent transcription before and after androgen stimulation

As we have reported previously, CDK1 and CDK9 phosphorylate AR at Ser81 by distinct mechanisms [6, 17]. Indeed, in AR transfected into 293T and COS1 cells grown in androgen‐depleted medium, nocodazole activated CDK1 (as marked by pCDK1‐T161) and led to pS81 expression in the absence of androgen (Fig. S6). Importantly, androgen‐stimulated pS81 can be further increased by co‐treatment with nocodazole. Similar observations were made in LNCaP cells co‐treated with nocodazole and various doses of androgen, and these findings were time‐dependent (Fig. S7). These observations support the co‐stimulation of pS81 by CDK1 and androgen, which acts through AR‐mediated recruitment of CDK9 to synthesize pS81 on the chromatin.

**Fig. 7:**
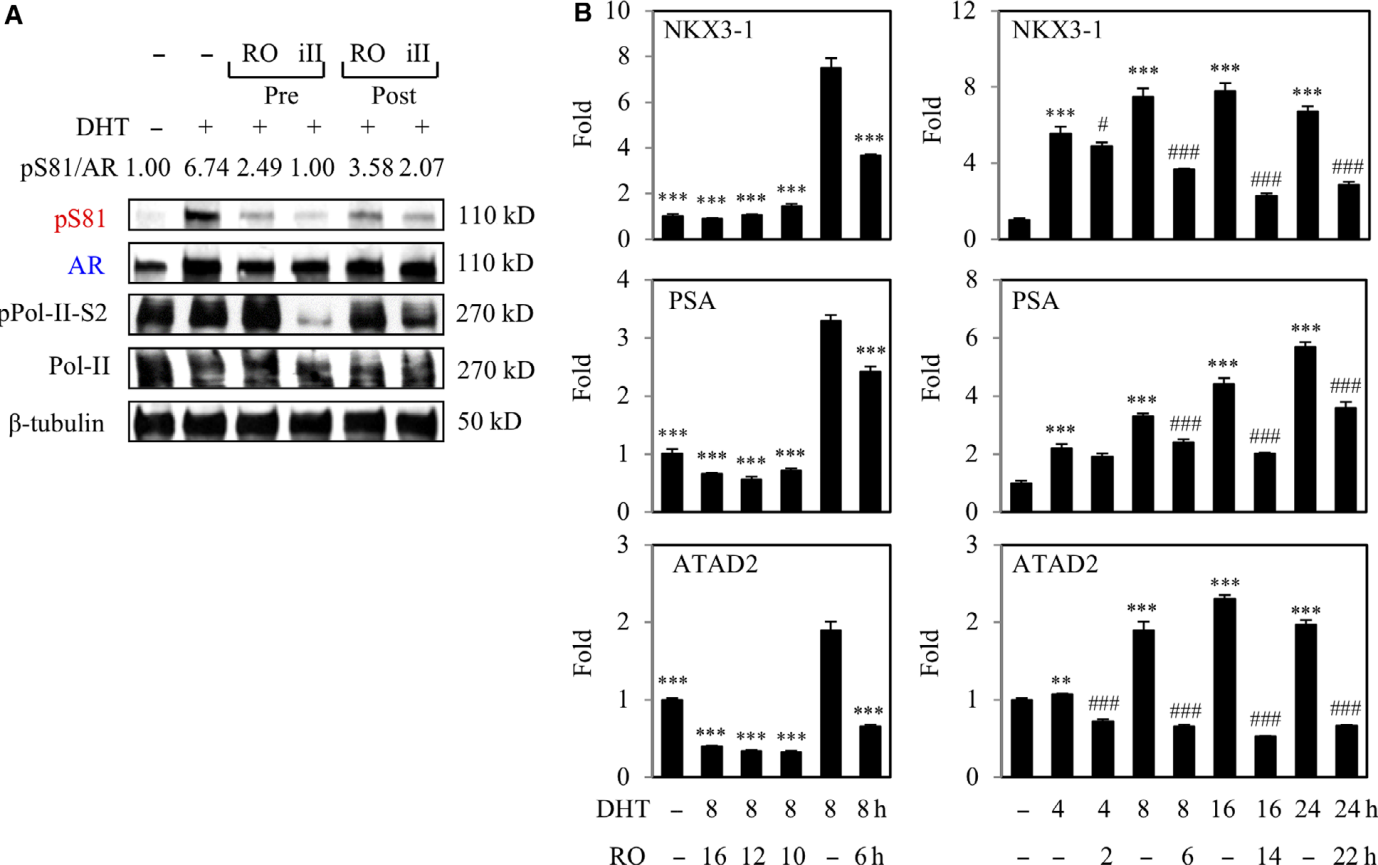
CDK1 activity is required to activate AR‐dependent transcription before and after androgen stimulation. (A) LNCaP cells in androgen‐depleted medium were treated with CDK1 and CDK9 inhibitors 2 h before (pretreatment) or after (post‐treatment) adding 10 nm DHT for 4 h. Total proteins were normalized for blotting. pS81 and AR signals were quantified using imagej and the normalized pS81/AR ratio is shown. (B) LNCaP cells in androgen‐depleted medium were treated with CDK1 inhibitor before or after adding 10 nm DHT for indicated treating times. Total RNA was subjected to RT‐PCR analysis. Each test includes three biologically independent replicates and results are presented as mean ± SD. Two‐tailed unpaired Student's *t*‐test was performed to calculate the statistical significance. For the left panels, DHT (8 h) only was used as control. For the right panels, different time‐points of DHT only were used as controls for DHT effects or compound effects. *P* < 0.05 was considered statistically significant: * (or ^#^) *P* < 0.05, ** (or ^##^) *P* < 0.01, *** (or ^###^) *P* < 0.001.

To validate the above findings functionally, we subjected LNCaP cells to androgen stimulation, together with pre‐ or post‐treatment with both CDK1 and CDK9 inhibitory compounds. As shown in Fig. 7A, pretreatment for 2 h with the CDK1 and CDK9 antagonists markedly repressed androgen‐stimulated pS81 expression. Of importance, upon a 2‐h DHT stimulation, post‐treatment with the CDK1 and CDK9 antagonists also substantially reduced androgen‐induced pS81 expression, although to a lesser extent than pretreatment did (Fig. 7A). These observations are consistent with the notion that both CDK1 and CDK9 are constantly catalyzing pS81 synthesis and are required for AR‐mediated transactivation. In addition, both pre‐ and post‐treatment with CDK1 compound attenuated androgen stimulation of PSA, NKX3.1 and ATAD2 gene expression, with more severely repressive impact occurring on the late androgen‐responsive *ATAD2* gene (Fig. 7B). Together, our findings are in line with the previous model [17] and with the above findings that pS81 functions to prime AR‐specific chromatin loci: the early androgen‐responsive gene loci can be initiated with the basal pS81 in the cells, whereas the elicitation of the late androgen‐responsive gene loci may require additional pS81 expression that takes time to accumulate within the cells. Mechanistically, both CDK1 and CDK9 are linked to AR transactivation via pS81 undergoing constant cellular re‐distribution.

### 3.7 CDK1 activity is maintained throughout the cell cycle in CRPC to sustain basal AR activation

**Fig. 8:**
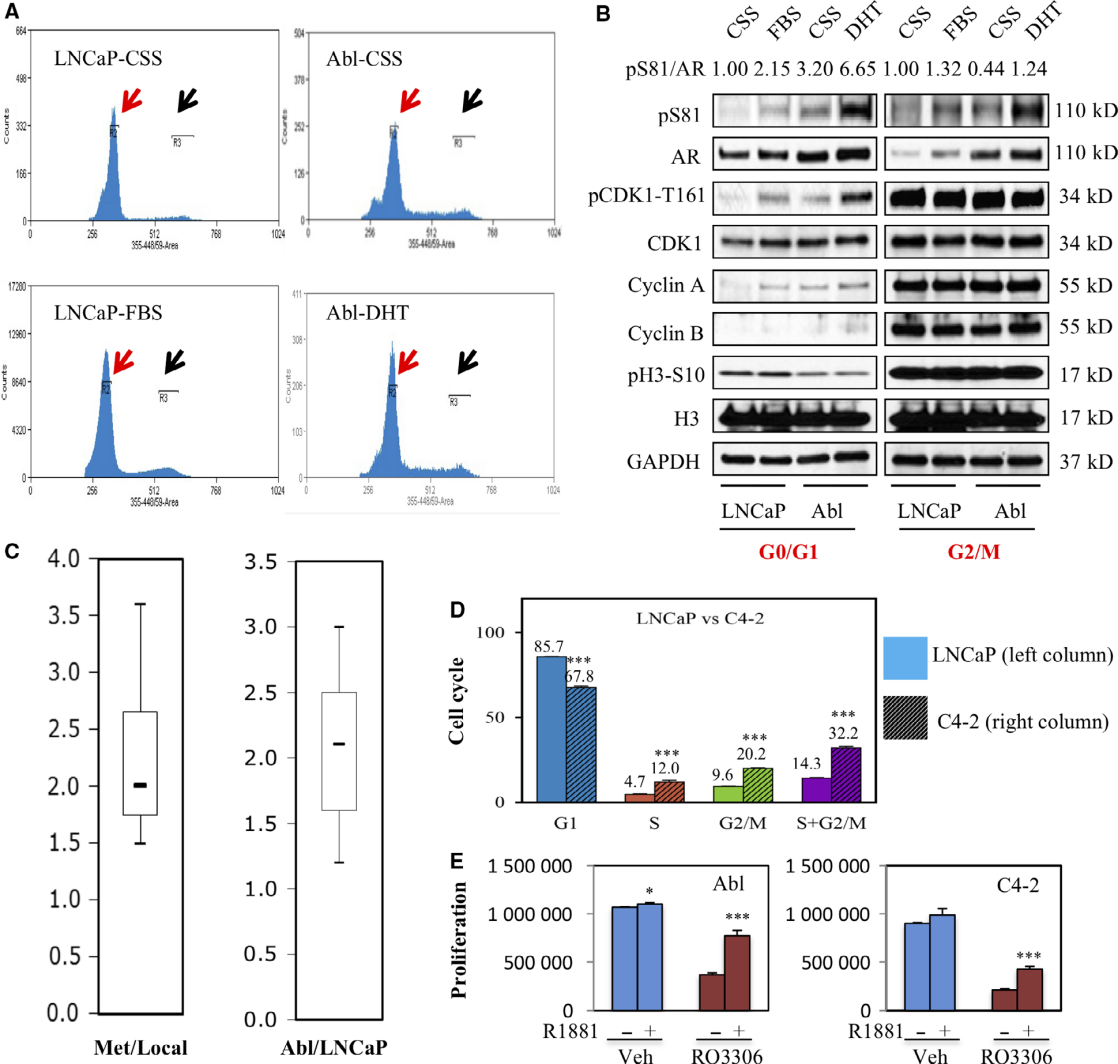
CDK1 activity is maintained throughout the cell cycle in CRPC to sustain basal AR activation. (A) Cell sorting diagrams of the LNCaP and Abl (cell culture developed CRPC cell line based on LNCaP) cell lines. LNCaP and Abl cells in indicated growth conditions were subjected to Hoechst staining and cell sorting. Red and black arrows indicate the gated G0/G1 (R2) and G2/M (R3) populations, respectively. (B) The above gated G0/G1 and G2/M populations were harvested and total proteins were normalized for blotting. pS81 and AR signals were quantified using imagej and the normalized pS81/AR ratio is shown. (C) Box‐plotting analysis for the relative expression of a 29‐gene signature (see Fig. S9) functionally enriched in DNA synthesis and cell cycle in the CRPC cell lines and clinical samples. The analyses (metastatic vs. primary PCa; Abl vs. LNCaP in androgen‐deprived condition) were based on global AR binding and Affymetrix microarray profiling [20, 21, 22, 23]. (D) LNCaP and its xenograft‐derived CRPC cell line (C4‐2) were grown in androgen‐deprived medium and subjected to PI staining and FACS analysis of cell cycle distribution. The distribution of indicated cell cycle phases was compared between LNCaP and C4‐2. (E) CRPC LNCaP‐Abl and C4‐2 cell lines were treated for 3 days with RO‐3306 (10 μm) and R1881 (10 nm) as indicated, followed by cell proliferation assay. Each test includes three biologically independent replicates and results are presented as mean ± SD. Two‐tailed unpaired Student's *t*‐test was performed to calculate the statistical significance; *P* < 0.05 was considered statistically significant: * *P* < 0.05, ** *P* < 0.01, *** *P* < 0.001.

We showed previously that CDK1 could increase pS81 to promote AR activity under androgen depletion [6, 9, 17]. The repressive effects on AR and cell proliferation by CDK compounds indicated that CRPC cells have CDK1 activity spread throughout the cell cycle, rather than just in a short interval during mitosis. To test this hypothesis, we grew LNCaP and CRPC Abl cells in steroid‐depleted (CDS) vs. androgen‐containing (FBS) media and then used fluorescence‐activated cell sorting (FACS) to isolate cells in G0/G1 and G2/M‐phases, respectively (Fig. 8A). We first observed that the G0/G1 Abl cells in CDS medium had substantial pS81, confirming that pS81 was not only present in cells during M‐phase (Fig. 8B). Using pT161 as an indicator of CDK1 activity, we then found CDK1 was activated in the G0/G1 Abl cells in CDS medium, which was correlated with increased cyclin A. Androgen could increase CDK1 activity in the G0/G1 Abl and LNCaP cells, in agreement with AR driving cell cycle progression, whereas the activity of CDK1 was similar and high in the G2/M LNCaP and Abl cells under both conditions (Fig. 8B). These results are consistent with CDK1 mediating the basal AR pS81 throughout the cell cycle in the Abl cells, and with the observations of rapid loss of pS81 in response to CDK1 inhibition [17].

As previously reported, global gene expression assessments showed that cell cycle genes are markedly increased in metastatic CRPC vs. primary androgen‐dependent PCa (Figs 8C, S8 and S9) [20]. The CRPC cell lines Abl and C4‐2 both expressed high levels of basal pS81 that are dependent on elevated CDK1 activity, consistent with increased G2‐M‐phase distribution in the cell cycle (Figs 8C,D, S8 and S9) [17]. These findings support an increase in CDK1 activity in CRPC and a strategy to inactivate AR under basal conditions by targeting CDK1.

We next asked whether CDK1 inhibition could restore androgen dependence to CRPC cells. For this purpose, Abl cells in CDS medium were stimulated with androgen (R1881, 10 nm) in the absence or presence of RO‐3306. In the vehicle ‐treated cells, R1881 did not stimulate cell proliferation (Fig. 8E), consistent with the known androgen‐refractory property of Abl [23]. In contrast, R1881 stimulated the proliferation of cells treated with RO‐3306. Treatment of the C4‐2 cells with RO‐3306 similarly rendered the cells responsive to R1881 stimulation (Fig. 8E). Together these results show that CDK1‐mediated pS81 can drive basal AR activity in CRPC cells and CDK1 activation is a mechanism to castration resistance.

## 4 Discussion

The profound effects of androgen on PCa biology and the robust spectrum of AR signature pathways attest to the importance of addressing AR re‐activation in CRPC. Consistent with its extraordinary structural features, Ser81 is phosphorylated time‐dependently and dose‐dependently by AR agonists but not antagonists [6, 11]. Previous studies have also shown an association of pS81 with AR nuclear localization and chromatin binding, and with AR‐p300 interaction [6, 8, 9, 10, 13, 16, 17]. Using ChIP assay to track pS81 signatures on the chromatin, here we confirmed that pS81 occupancy to AR‐mediated loci is intrinsically linked to androgen stimulating different subsets of AR‐responsive genes. We further conducted pS81‐specific vs. AR‐specific ChIP‐Seq analyses to disclose an unusual enrichment of pS81 at AR‐activated promoters, along with pTEFb and activated RNA pol II that occupy the Enh‐Pro loop for transactivation. Significantly, using a 3C‐based ChIP assay, we verified that Ser81‐phosphorylated AR was indeed enriched in the PSA Enh‐Pro looping complex. These observations together with the ReChIP findings on pS81 as a partner of BRD4/pTEFb in the Enh‐Pro vicinity are all in accordance with the coupling of pS81 (but not total AR) to the general transactivation machinery.

It is a seminal observation that Ser81‐phosphorylated and ‐unphosphorylated AR have differential binding profiles. The underlying mechanisms could be attributed to phosphorylation‐mediated conformation change and/or formation distinct complexes; an alternative explanation is that only a small fraction of total AR is looped at the Enh‐Pro loci, where it is phosphorylated by pTEFb/CDK9 in proximity to the promoter. Similar to the androgen‐dependent and chromatin‐bound pS81 transactivation mediated by CDK9, under basal condition we identified the dependence of pS81 expression on CDK1 that is hyperactivated in CRPC cells. An enrichment of pS81 nuclear distribution is consistent with reports that S81‐phosphorylated AR is preferentially distributed to the nucleus in PCa [9, 11, 15]. Mechanistically, the nuclear‐enriched pS81 can prime the target gene locus and confer hypersensitivity of AR to various ligands, thus contributing to castration resistance. Indeed, we have reported that in CRPC cells the basal pS81 was predominantly mediated by CDK1 and was not blocked by the AR antagonists [17]. In this report, we further determined that CDK1 functions in the soluble cell compartments to maintain the basal pS81 and AR transactivation, supporting the CDK1‐dependence of AR transcription under androgen‐depleted status. Consistently, in CRPC cells, AR transactivation is effectively attenuated by a CDK1 inhibitor that can restore androgen responsiveness, providing a rationale to target CDK1.

## 5 Conclusion

Collectively, our report could guide future studies on the potential mechanisms and functions of pS81 chromatin binding and on additional AR phosphorylation sites or other post‐translational modifications that may also have distinct binding profiles and functional significance. Here our observations delineated the circumstances in which CDK1 and CDK9 activate AR: both S81 kinases contribute the S81 phosphorylation of AR that is dynamically distributed to various soluble compartments and the chromatin. Significantly, our findings uncovered extraordinary pS81 chromatin occupancy profiling: its looping in the AR‐activated Enh‐Pro loci and its coupling to the general transcription machinery. These findings together rationalize the strategy to target these pS81 kinases, alone or in conjunction with direct AR antagonists in CRPC patients.

## Conflict of interest

The authors declare no conflicts of interest.

## Author contributions

XTG and JQL performed all experimental tests. LYW, ZYZ, PHY and JXW participated in ChIP and re‐ChIP tests. YG participated in cell sorting, blotting and ChIP analyses. FM participated in Bioinformatics analysis. CC, HHY and OV participated in IHC tests. SGW, TW and JHL participated in the experimental design. SC and XML were involved in all aspects of the study.

### Peer Review

The peer review history for this article is available at https://publons.com/publon/10.1002/1878‐0261.12968.

## Supporting information


**Fig. S1**. AR‐regulated genes were grouped into time‐dependent activation subsets.

**Fig. S2**. AR‐regulated genes were grouped into time‐dependent activation subsets.

**Fig. S3**. Characteristics of AR and pS81 chromatin binding profiling.

**Fig. S4**. Basic information on 3C target region and PCR primers.

**Fig. S5**. Validation of 3C‐ChIP nest‐PCR products by sequencing and annotation.

**Fig. S6**. A repeat reChIP test and a re‐analysis of the reChIP tests.

**Fig. S7**. CDK1 synergizes androgen to co‐stimulate pS81 expression.

**Fig. S8**. Gene set enrichment analysis (GSEA) study indicated shared gene overexpression signatures between PCa cell lines and clinical samples.

**Fig. S9**. Overexpression of DNA repair and G2‐M genes in CRPC cells.

## Data Availability

The data that support the findings of this study are openly available in GEO datasets at https://www.ncbi.nlm.nih.gov/gds: reference numbers GSE166192, GSE32269, GSE11428, GSE31410 and GSE32356.
