# Supplementary figures and images for: Phosphorylation of the androgen receptor at Ser81 is co‐sustained by CDK1 and CDK9 and leads to AR‐mediated transactivation in prostate cancer
Source: Mol Oncol. 2021 May 3;15(7):1901–20. doi: 10.1002/1878-0261.12968 (PMC8253089; doi:10.1002/1878-0261.12968)

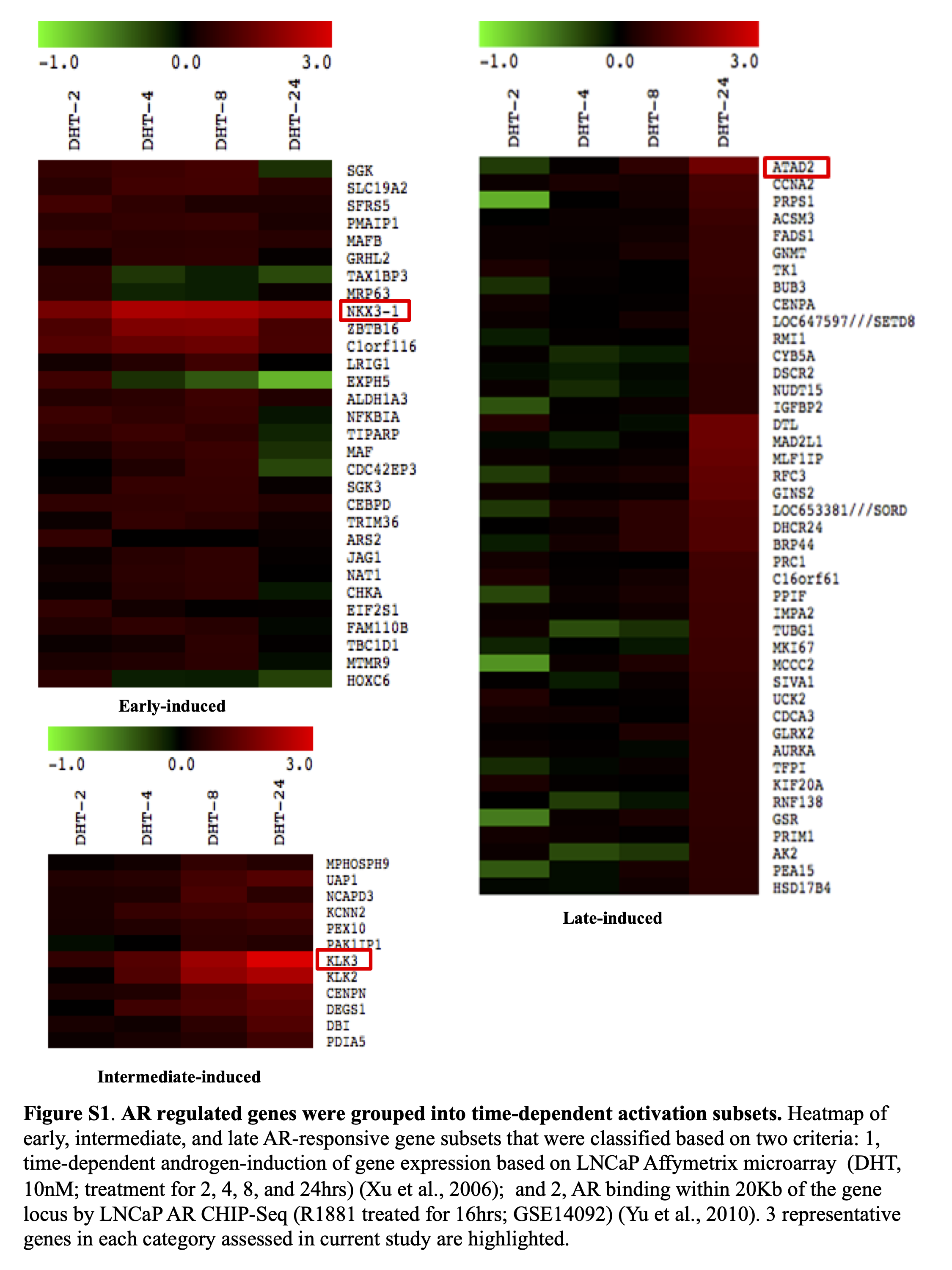

Supplement: Supplementary file 1 — Fig. S1. AR‐regulated genes were grouped into time‐dependent activation subsets. [file MOL2-15-1901-s008.tiff]

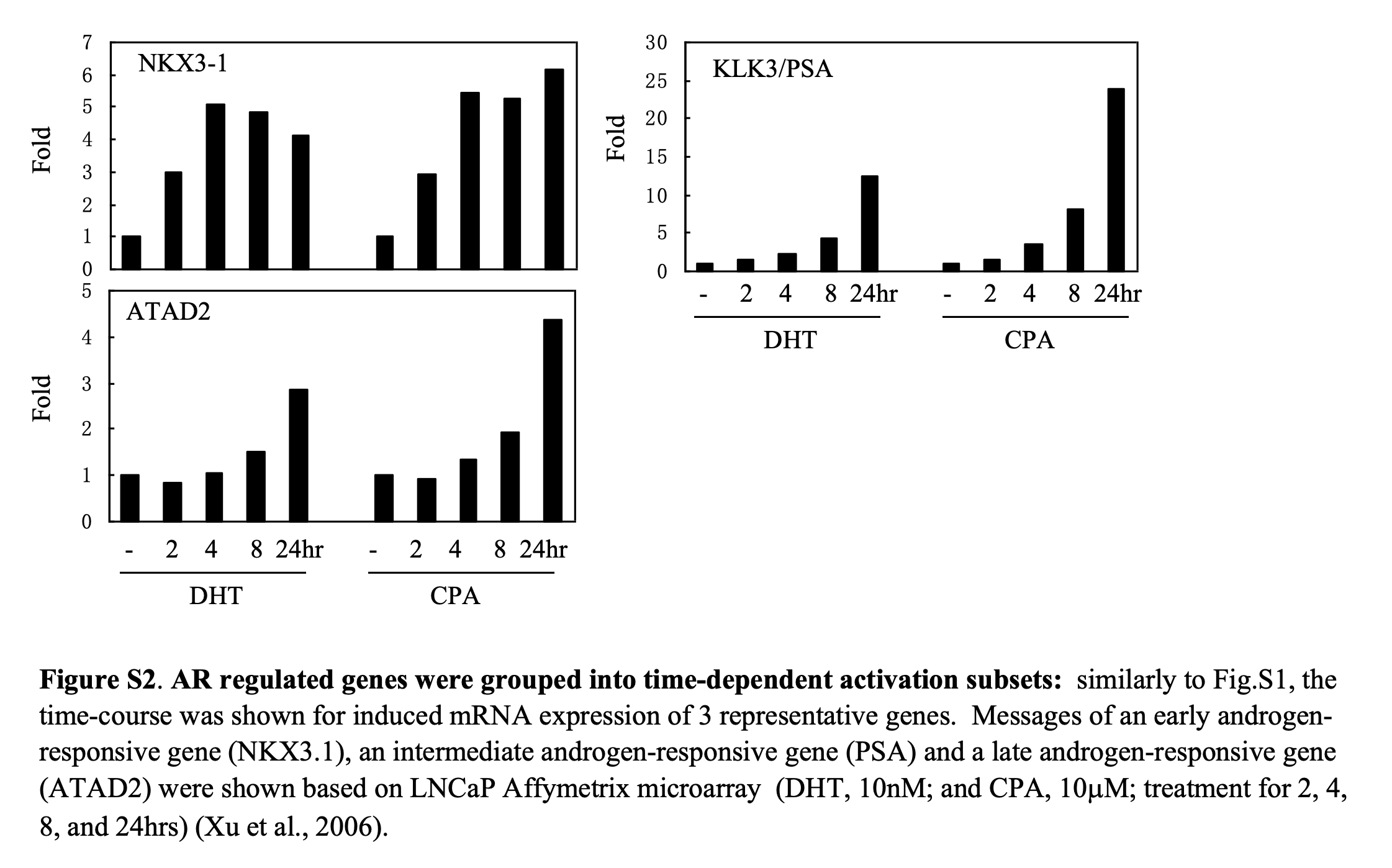

Supplement: Supplementary file 2 — Fig. S2. AR‐regulated genes were grouped into time‐dependent activation subsets. [file MOL2-15-1901-s007.tiff]

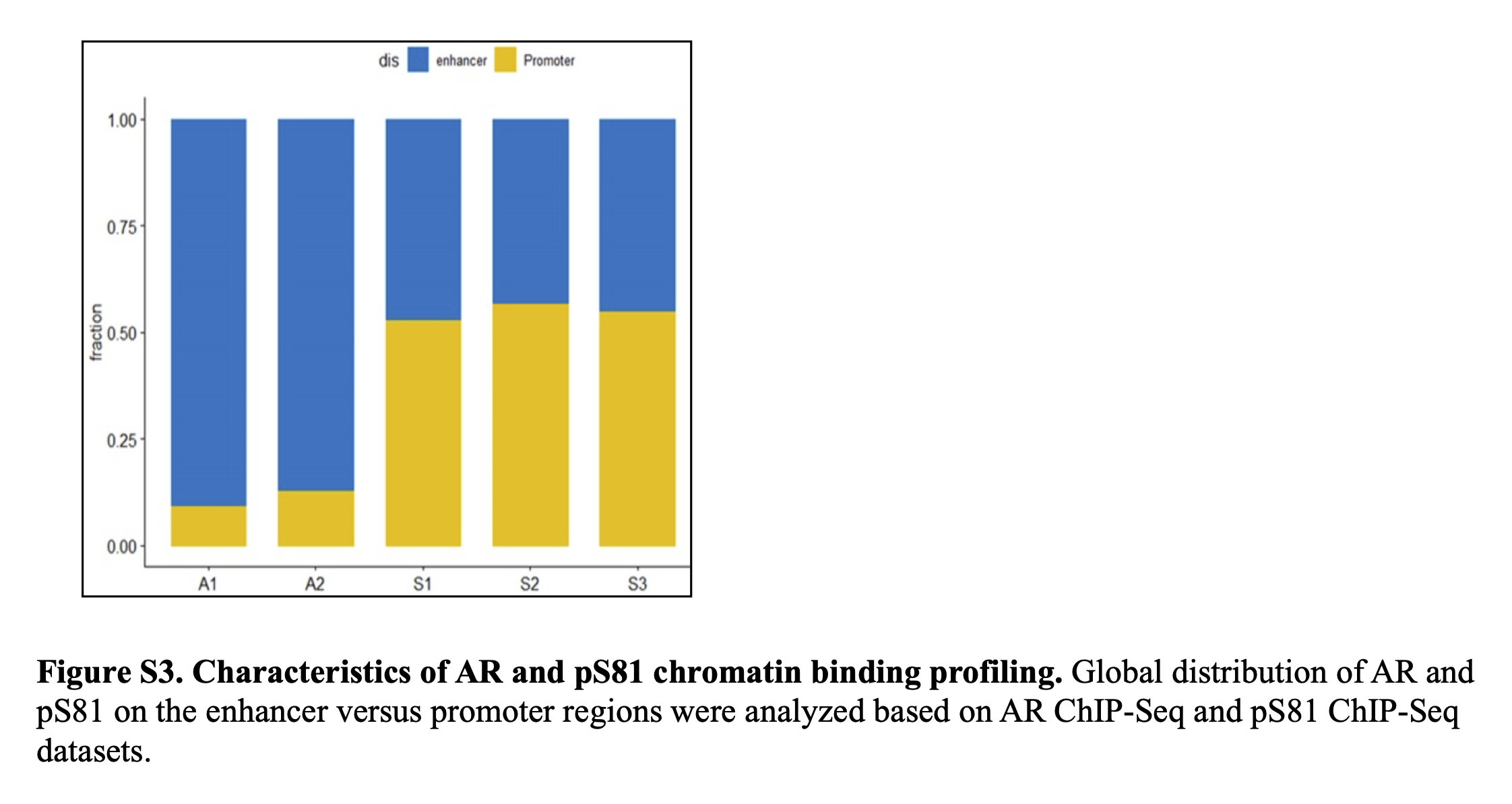

Supplement: Supplementary file 3 — Fig. S3. Characteristics of AR and pS81 chromatin binding profiling. [file MOL2-15-1901-s005.tiff]

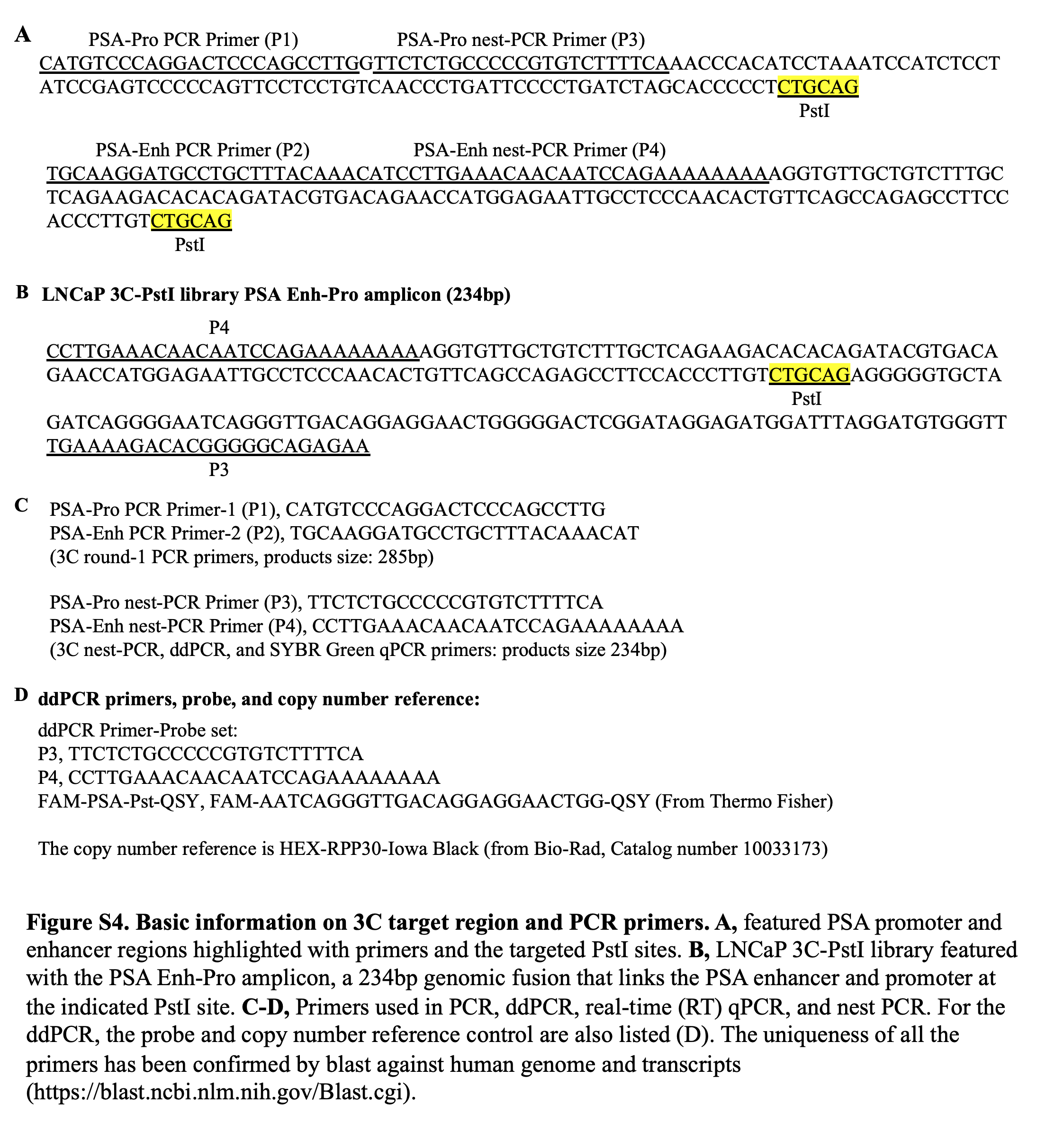

Supplement: Supplementary file 4 — Fig. S4. Basic information on 3C target region and PCR primers. [file MOL2-15-1901-s006.tiff]

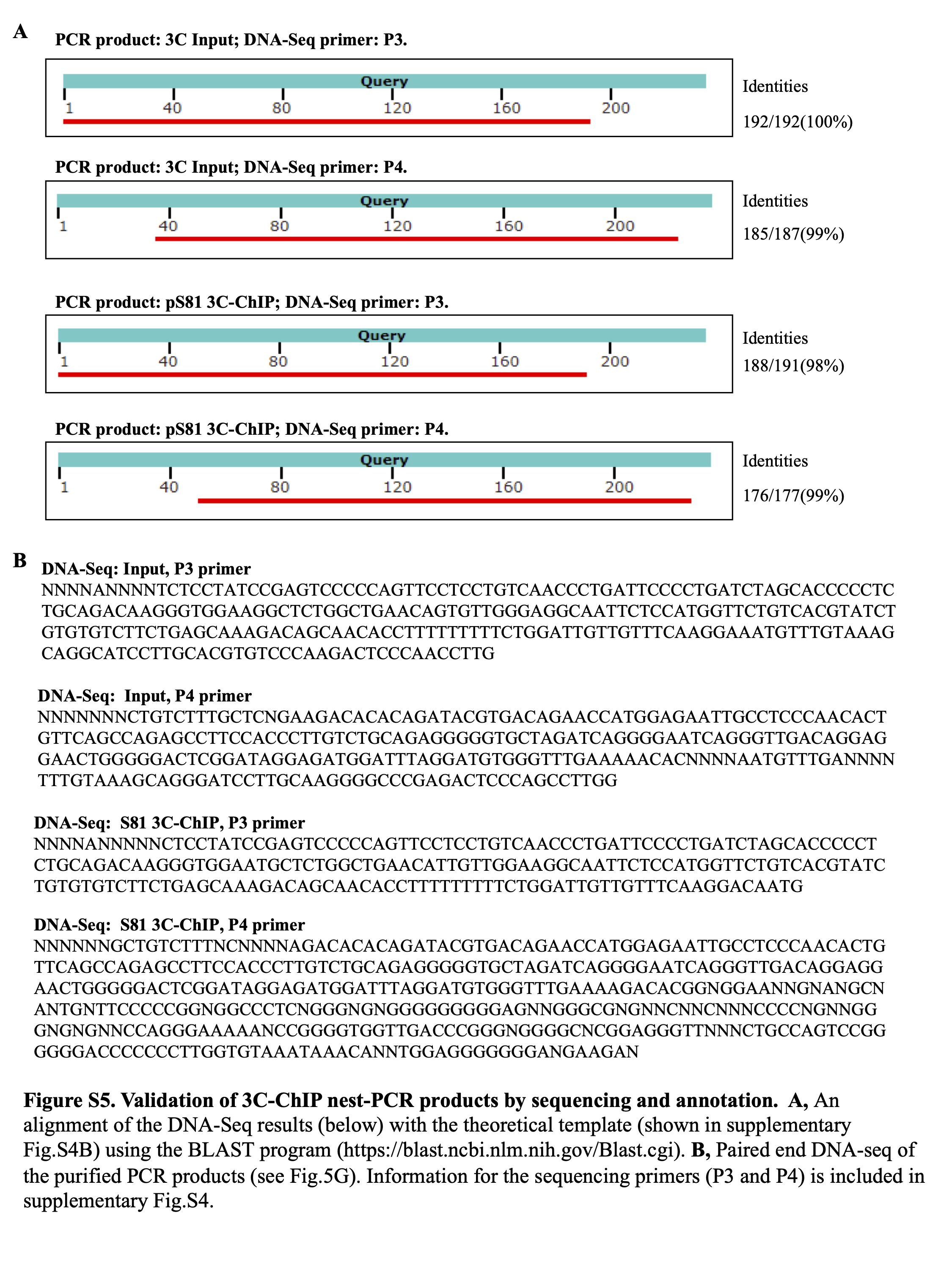

Supplement: Supplementary file 5 — Fig. S5. Validation of 3C‐ChIP nest‐PCR products by sequencing and annotation. [file MOL2-15-1901-s001.tiff]

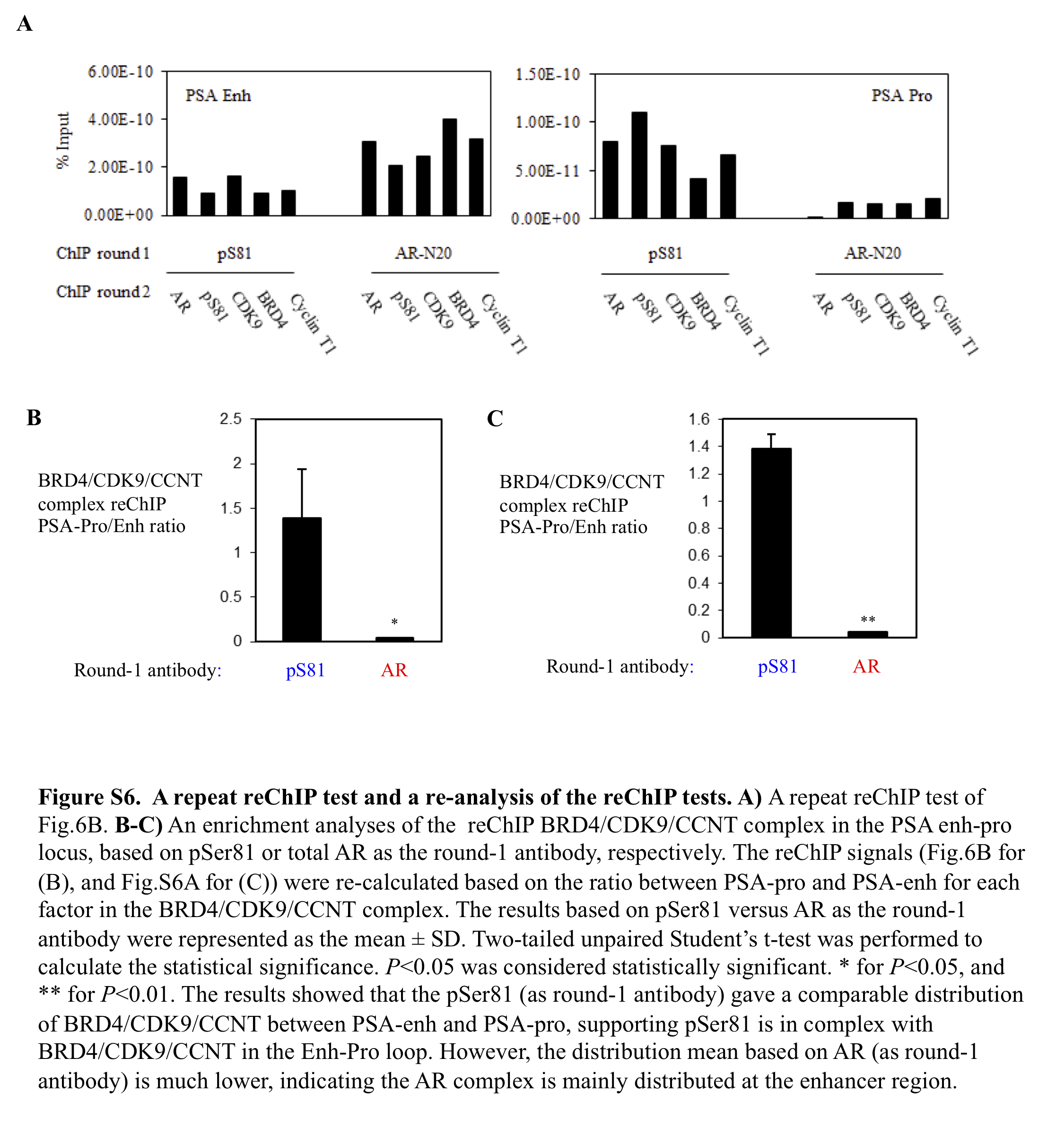

Supplement: Supplementary file 6 — Fig. S6. A repeat reChIP test and a re‐analysis of the reChIP tests. [file MOL2-15-1901-s002.tiff]

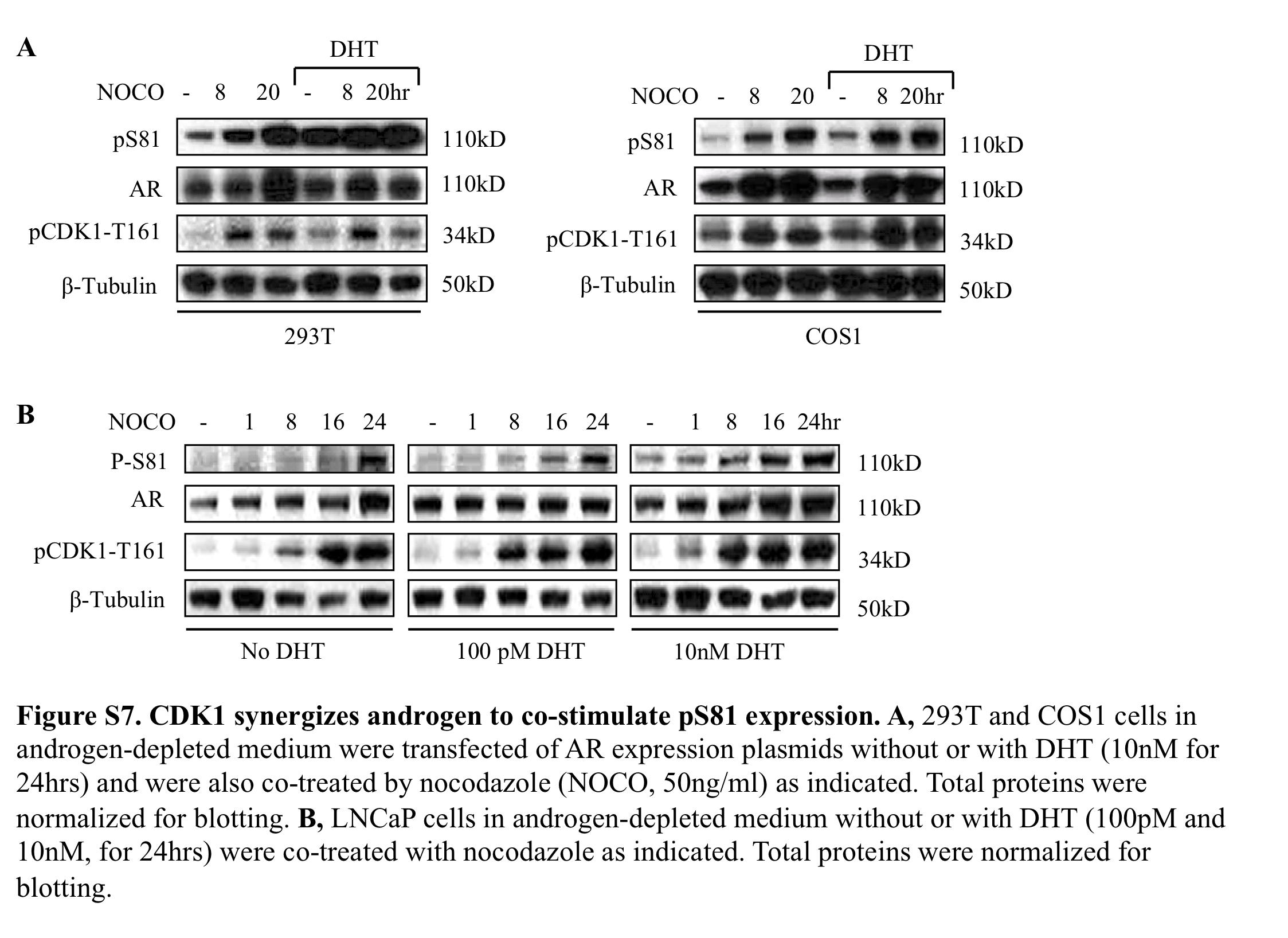

Supplement: Supplementary file 7 — Fig. S7. CDK1 synergizes androgen to co‐stimulate pS81 expression. [file MOL2-15-1901-s003.tiff]

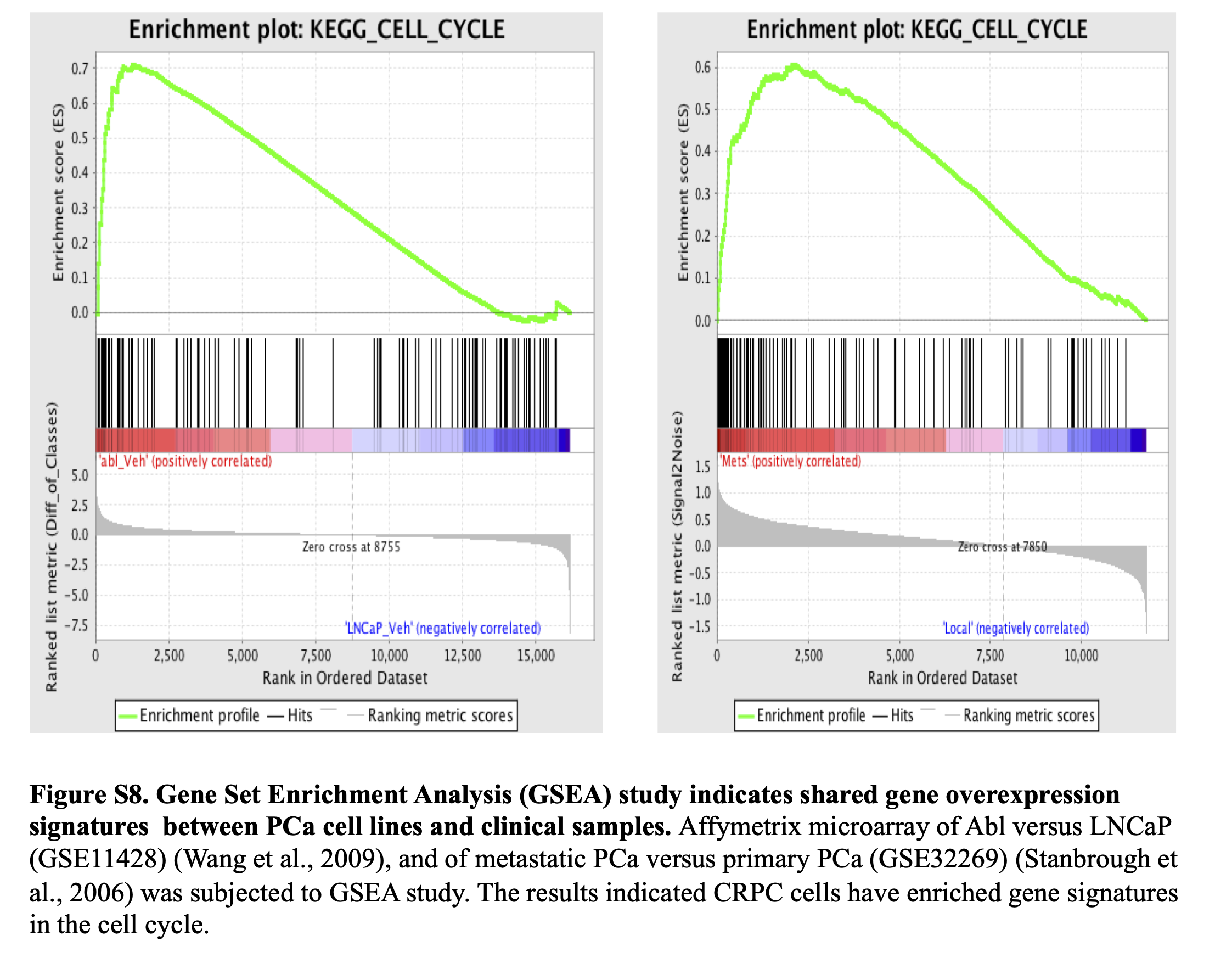

Supplement: Supplementary file 8 — Fig. S8. Gene set enrichment analysis (GSEA) study indicated shared gene overexpression signatures between PCa cell lines and clinical samples. [file MOL2-15-1901-s010.tiff]

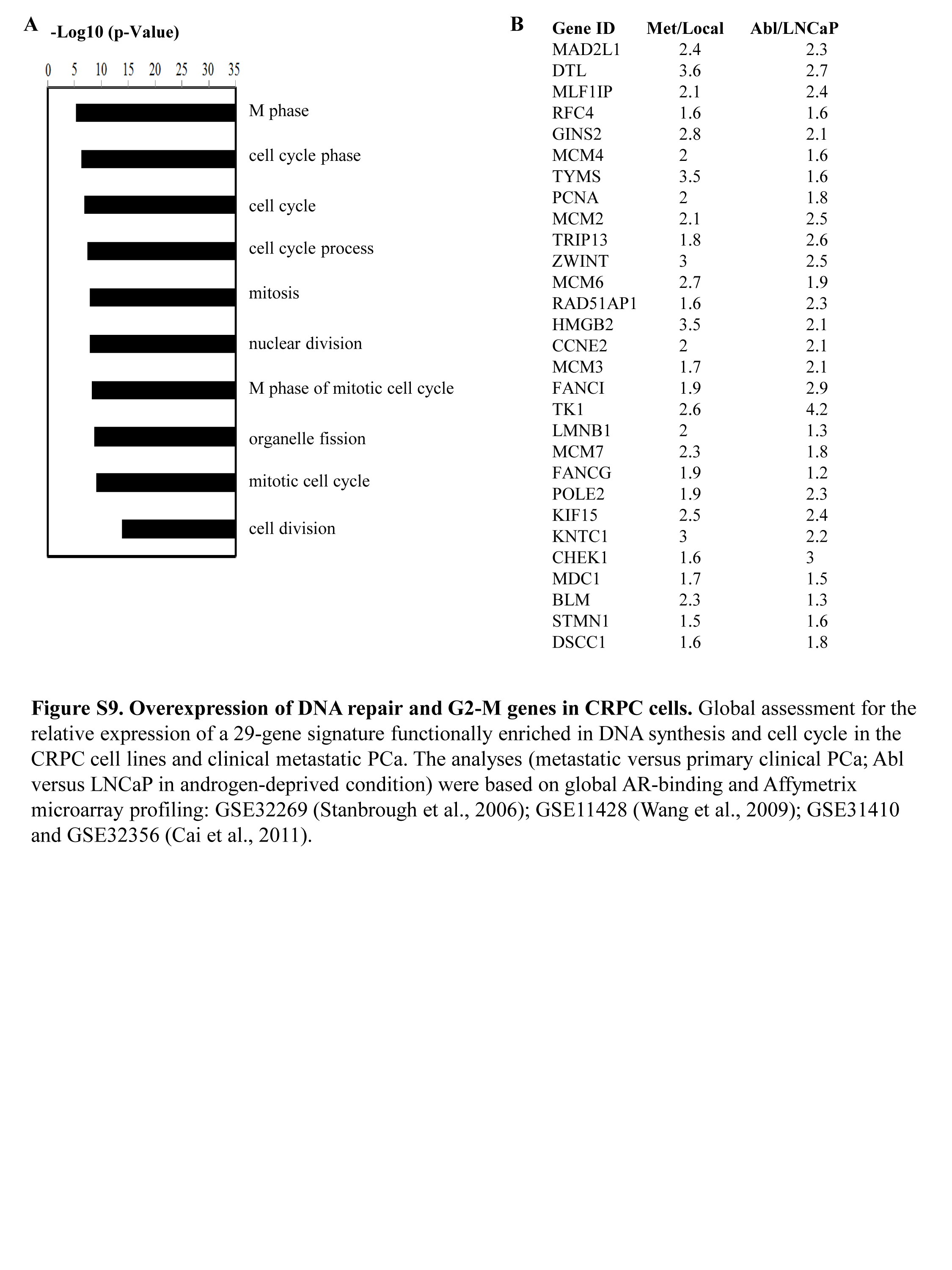

Supplement: Supplementary file 9 — Fig. S9. Overexpression of DNA repair and G2‐M genes in CRPC cells. [file MOL2-15-1901-s004.tif]

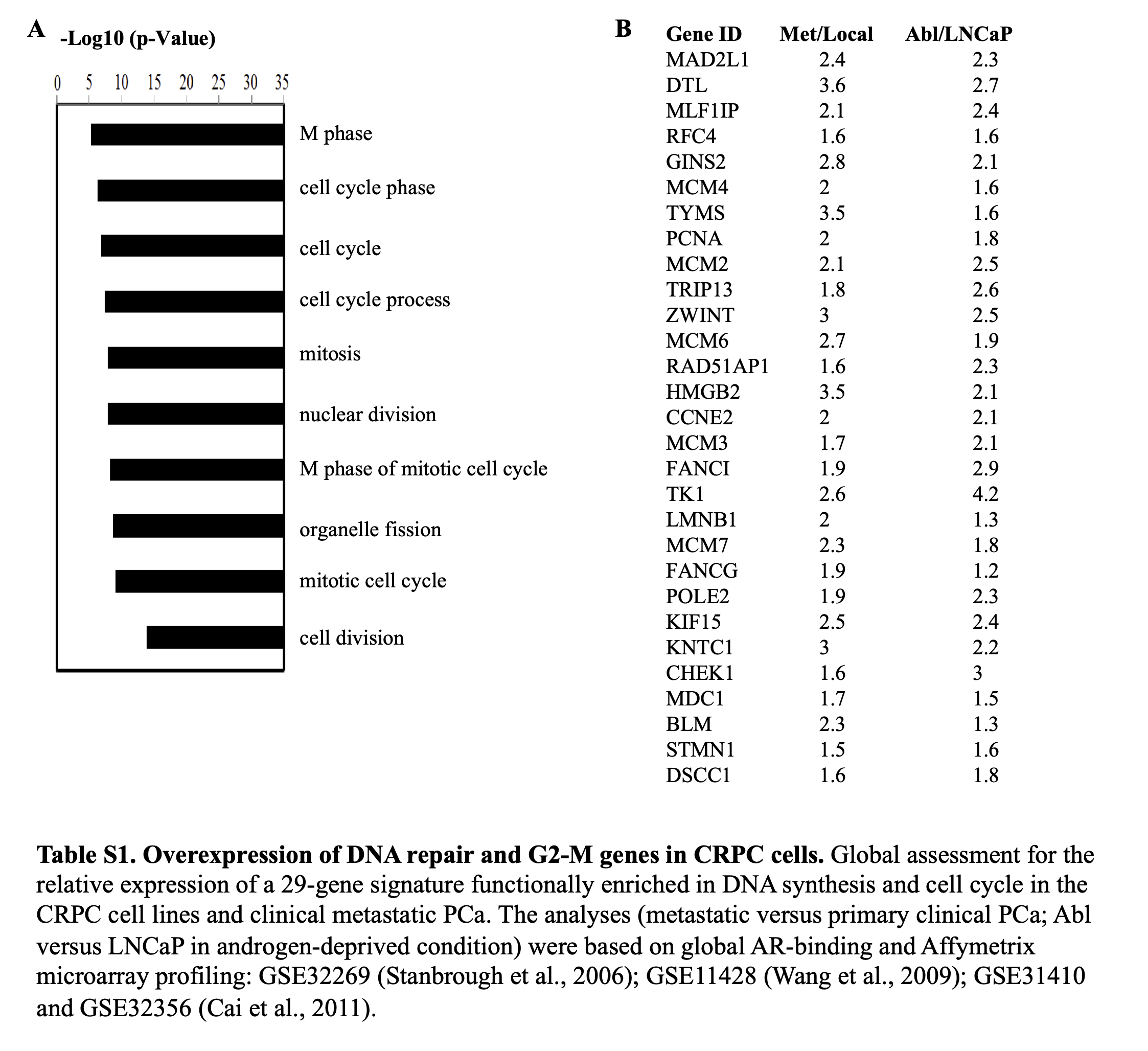

Supplement: Supplementary file 10 [file MOL2-15-1901-s009.tiff]
